# Efficacy and safety of zuranolone in major depressive disorder: a meta-analysis of factor effect and dose-response analyses

**DOI:** 10.1016/j.eclinm.2023.102308

**Published:** 2023-11-16

**Authors:** Yu-Wei Lin, Yu-Kang Tu, Kuo-Chuan Hung, Chih-Sung Liang, Ping-Tao Tseng, Pao-Yen Lin, Edward Chia-Cheng Lai, Chih-Wei Hsu

**Affiliations:** a Department of Psychiatry, Kaohsiung Chang Gung Memorial Hospital and Chang Gung University College of Medicine, Kaohsiung, Taiwan; b Institute of Health Data Analytics & Statistics, College of Public Health, National Taiwan University, Taipei, Taiwan; c Department of Dentistry, National Taiwan University Hospital, Taipei, Taiwan; d Department of Anesthesiology, Chi Mei Medical Center, Tainan, Taiwan; e Department of Psychiatry, Beitou Branch, Tri-Service General Hospital, National Defense Medical Center, Taipei, Taiwan; f Department of Psychiatry, National Defense Medical Center, Taipei, Taiwan; g Prospect Clinic for Otorhinolaryngology & Neurology, Kaohsiung, Taiwan; h Institute of Biomedical Sciences, National Sun Yat-sen University, Kaohsiung, Taiwan; i Department of Psychology, College of Medical and Health Science, Asia University, Taichung, Taiwan; j Institute of Precision Medicine, National Sun Yat-sen University, Kaohsiung City, Taiwan; k School of Pharmacy, Institute of Clinical Pharmacy and Pharmaceutical Sciences, College of Medicine, National Cheng Kung University, Tainan, Taiwan

**Keywords:** Depression, DRMA, MDD, Postpartum, SAGE-217, Zuranolone

## Abstract

**Background:**

Zuranolone is recognised as a promising antidepressant agent. Our study aimed to investigate the efficacy and safety of zuranolone in treating major depressive disorder (MDD).

**Methods:**

A systematic review was conducted by searching major databases from inception to August 20, 2023 (INPLASY: 202360087). A meta-analysis was performed by using a random-effects model to calculate effect sizes, expressed as standardised mean differences (SMDs) and odds ratios (ORs) with 95% confidence intervals (CIs). The primary outcome was improvement in depressive symptoms, while secondary outcomes included response and remission rates of depression, improvement in anxiety symptoms, incidence of dropouts, and any side effects. We conducted subgroup analyses for general MDD and postpartum-onset MDD and a dose-response meta-analysis to estimate the relationship between zuranolone dose and outcomes.

**Findings:**

The study included seven randomised control trials involving 1789 patients. Zuranolone reduced depressive symptoms (SMD = −0.37, 95% CIs = −0.51 to −0.23), increased response rate (OR = 2.06, 95% CIs = 1.48–2.85) and remission rate (OR = 2.04, 95% CIs = 1.38–3.02), and reduced anxiety symptoms (SMD = −0.26, 95% CIs = −0.39 to −0.14). Furthermore, zuranolone-treated patients experienced more side effects than those in the control group (OR = 1.40, 95% CIs = 1.10–1.78), although dropout rate did not significantly differ between the two groups (OR = 1.13, 95% CIs = 0.85–1.49). According to the dose-response meta-analysis, zuranolone could effectively improve depression and anxiety at increasing doses up to a maximum daily dose of 30 mg; however, side effects increased with doses exceeding 30 mg. Based on subgroup analyses, zuranolone showed greater efficacy in treatment of postpartum-onset MDD than general MDD, but the difference did not reach statistical significance.

**Interpretation:**

Our findings suggested that zuranolone is effective in alleviating depression and anxiety. Nevertheless, there is a potential risk of adverse effects. Given its therapeutic efficacy and risk of side effects, a daily dose of 30 mg appears to be the optimal choice.

**Funding:**

10.13039/501100004606Chang Gung Medical Foundation.

**Research in context**

**Evidence before this study**

We searched the PubMed, EMBASE, Cochrane CENTRAL, Web of Science, ProQuest, Clinical Key, ScienceDirect, ClinicalTrials.gov database, and grey literature from database inception until August 20, 2023. Several randomised controlled trials have investigated the efficacy and safety of zuranolone in treatment of treatment of major depressive disorder. However, considering its efficacy and safety, a comprehensive integration of these results and optimal dosing recommendations is still lacking.

**Added value of this study**

Our meta-analysis provides evidence supporting the efficacy of zuranolone in reducing depressive symptoms, improving response and remission rates, and alleviating concurrent anxiety symptoms in patients with major depressive disorder compared to placebo. However, although dropout rate was similar between the zuranolone and placebo groups, caution should be exercised regarding the potential side effects. Considering the dose-response relationship, our findings suggest that a daily dosage of 30 mg of zuranolone produces favourable outcomes, notably reductions in symptoms of depression and anxiety. Furthermore, the dropout rate is comparable to that of the placebo.

**Implications of all the available evidence**

Our findings suggested that a daily dose of zuranolone of 30 mg could be considered an appropriate and effective treatment option for major depressive disorder, as it offers a favourable balance between efficacy and safety.

## Introduction

People with major depressive disorder (MDD) may experience reduced quality of life and impaired social functioning.^1^ Despite the prevalence of available antidepressant treatments, nearly one-third of patients remain poorly responsive to current antidepressants. For example, the Sequenced Treatment Alternatives to Relieve Depression (STAR∗D) trial revealed that only 37% of individuals with MDD achieved remission during the first-line treatment.^2^ Furthermore, the odds of achieving remission were found to decrease with the subsequent line of treatment.^3^ Even after four lines of treatment, the remission rate was only achieved in approximately two-thirds of the total population.^2^ Typically, standard-of-care antidepressants take two weeks or longer to exhibit effects and frequently require long-term administration, contributing to poor compliance and creating a vicious cycle.^2^ Common side effects, such as insomnia, weight gain, and sexual dysfunction, can further impact treatment adherence, leading to dose reduction, interruption, or nonadherence.^4^ Therefore, there is an urgent need to develop new antidepressants for treating MDD as a tool for clinicians.

Zuranolone has been in development as a novel antidepressant since 2017.^5^ It is a neurosteroid, allopregnanolone, that acts as a positive allosteric modulator of the extrasynaptic γ-aminobutyric acid (GABA) receptor, which distinguishes it from benzodiazepines. Studies have consistently observed low concentrations of GABA in the plasma and cerebrospinal fluid,^6^ reduced cortical GABA levels, and decreased GABAergic interneurons in individuals with depression.^7^ The lack of inhibitory action associated with these alterations may contribute to the imbalance in brain networks observed in depression, and zuranolone could correct this imbalance. Moreover, the abundant distribution of allopregnanolone in the amygdala,^8^ along with its normalisation following the remission of depression,^9^ provides further evidence of the potential efficacy of this compound. Additionally, the presence of zuranolone is highlighted by another US Food and Drug Administration (FDA)-approved allopregnanolone product, brexanolone, indicated for treating postpartum depression and administered via continuous intravenous infusion over 60 h.^10^

Several randomised controlled trials (RCTs) have recently focused on the use of zuranolone to treat MDD, particularly in postpartum depression,^11^ suggesting that zuranolone may improve symptoms of depression and even anxiety,^11^^,^^12^ although several side effects have been documented.^13^ To the best of our knowledge, no meta-analysis has been conducted to collate the most recent medical evidence on the efficacy and safety of zuranolone. Furthermore, these RCTs explored different groups (general MDD^14^ and postpartum-onset MDD^11^) and used different dosages (20 mg/30 mg/50 mg).^13^^,^^15^ Therefore, whether the observed outcomes or side effects are related to the specific groups or dosages examined remains unclear.

In the current study, we aimed to investigate RCTs on zuranolone using a meta-analysis approach, focusing on its efficacy in treating depression and anxiety, including its safety concerning dropout rates and side effects. Furthermore, we aimed to explore the factors that might influence these results, such as population characteristics and dosing considerations.

## Methods

### Search strategy and study selection

This meta-analysis was conducted in accordance with the Preferred Reporting Items for Systematic Reviews and Meta-Analyses 2020 statement (PRISMA 2020, eTable 1),^16^ following protocols registered at the International Platform of Registered Systematic Review and Meta-Analysis Protocols (INPLASY 202360087). The Institutional Review Board of the Chang Gung Memorial Hospital reviewed the study protocol and waived the need for ethical approval (no. 202200827B1).

A systematic literature search was conducted across PubMed, EMBASE, Cochrane CENTRAL, Web of Science, ProQuest, Clinical Key, ScienceDirect, ClinicalTrials.gov database, and grey literature from database inception until August 20, 2023, using keywords such as (depress∗ OR “affective” OR “mood”) AND (“zuranolone” OR “SAGE-217” OR “S-812217”). No language and region restrictions were applied. Details of the search strings and the database used are listed in eTable 2. Additionally, the pharmaceutical company that manufactures zuranolone was contacted for potential unpublished data. The formulated PICO (Patient, Intervention, Comparison, and Outcome) questions for our study were as follows: (1) Patient: Individuals diagnosed with a major depressive episode of MDD; (2) Intervention: Administration of a complete regimen of zuranolone; (3) Comparison: Placebo or other antidepressants; and (4) Outcome: Alterations in the severity of depression and anxiety, and dropout and adverse event incidence rates.

Inclusion criteria were as follows: (1) RCTs with placebo or antidepressants as the control group; (2) Diagnosis of MDD, with or without postpartum onset, according to accepted diagnostic criteria (e.g., Diagnostic and Statistical Manual of Mental Disorders); and (3) quantification of depression severity using a valid scale, both pre- and post-zuranolone regimen administration. Exclusion criteria were as follows: (1) non-randomised trials, including literature reviews and case studies or series; (2) letters to the editor or editorial commentary; and (3) RCTs lacking a placebo or antidepressant control group. In cases where the data were duplicated (e.g., multiple publications resulting from the same data source), only reports that provided the most comprehensive information and were based on larger sample sizes were selected. After screening titles and abstracts, we performed a full-text review to identify eligible studies reporting the efficacy and safety of zuranolone for treating depression. Two authors (YWL and CWH) conducted the review and study selection independently, with a third author (PTT) consulted to resolve any discrepancies during the full-text review.

### Data extraction and quality assessment

Two authors (YWL and CWH) extracted relevant data, including the year of publication (or year of clinical trial registration), names of authors, demographic characteristics (age, sex, diagnostic criteria for depression, and baseline depression severity), and study design elements (dosing regimen and duration of treatment), from each eligible study. Given that most studies assessing zuranolone employed the 17-item Hamilton Rating Scale for Depression (HAMD-17) as their primary evaluation tool,^11^^,^^14^^,^^15^ our focus for depression severity was centred on alterations in the HAMD-17 score. If the included studies did not employ the HAMD-17 for assessing depression, we selected the primary outcome scale utilised in the relevant studies to assess depression as an alternative, such as the 21-item HAMD or Montgomery Asperger Depression Rating Scale (MADRS).^17^ Furthermore, we recorded the response and remission rates of depressive symptoms as secondary outcomes, respectively, defined as a reduction in depression scores by more than 50% and a fall in depression scores below a specific cut-off point. In cases where depressive symptoms were assessed using the HAMD-17, a score of 7 was classified as remission.^11^^,^^13^^,^^15^ Additionally, our secondary outcome assessed changes in patient anxiety levels. In most studies, except for one study (National Library of Medicine [NLM], NCT003771664) that did not record participants’ anxiety severity, the Hamilton Anxiety Rating Scale (HAM-A) or the anxiety subscale of the HAMD-17 were typically employed as evaluative tools for anxiety symptoms.^12^^,^^15^ Other secondary outcomes included dropouts and side effects. Dropouts were defined as participants who did not complete the study for any reason. Regarding side effects, we included any treatment-emergent adverse events recorded during the study, regardless of their severity. Dropout and side effect rates were calculated by dividing the number of dropouts or side effects, respectively, by the total number of random participants.

The risk of bias among the included studies was independently determined by two authors (YWL and CWH), following the risk of bias tool in the Cochrane Handbook.^18^ Any discrepancies were resolved through consensus discussions involving a third author (PTT).

### Data synthesis and statistical analysis

In the meta-analysis of continuous variables, such as the severity of depression or anxiety, we calculated effect sizes based on between-group differences (treatment and placebo) in changes in depression and anxiety scores (pre- and post-value), utilising the standardised mean difference (SMD) with 95% confidence intervals (CIs). If all included studies used a consistent measurement tool, such as HAMD-17, we conducted a sensitivity analysis using the mean difference (MD) as a measure. For dichotomous outcomes, such as depression response or remission rate, incidence of side effects, and dropout rate, we used the odds ratio (OR) as a measure. Given the potential heterogeneity among various studies, we conducted a meta-analysis using a random-effects model. Moreover, we performed a leave-one-out sensitivity analysis to identify RCTs that might be a potential source of heterogeneity. Given that we were interested in the potential differences in the effect of zuranolone on MDD and postpartum-onset MDD, we performed a subgroup analysis specifically for these two diagnoses. Additionally, we conducted a meta-regression analysis to identify potential confounding factors, including pre-treatment depression severity, age, and sex.

We also referenced our previous research to conduct a one-stage, random-effects, dose-response meta-analysis.^19^ We defined the dose for each zuranolone category based on the therapeutic dose utilised in the included studies (i.e., 0 mg for placebo; 30 mg for zuranolone). To discern the relationship between exposure dose and outcomes, we applied the methodologies proposed by Orsini, which facilitated the exploration of nonlinear trends. In our analysis, restricted cubic splines with three knots at fixed percentiles (10, 50, and 90%) of the distribution of zuranolone in the included studies were used to model the dose-response relationship.^20^ The model was then estimated using a generalised least squares estimator accounting for the correlation between effect sizes (SMD or OR) in each study.^21^ The choice of three knots and fixed percentiles of 10, 50, and 90% were based on recommendations from previous research^22^ and the limited dose categories of zuranolone observed in the current studies. Additionally, we performed a sensitivity analysis at fixed percentiles (10, 75, and 95%) of the zuranolone distribution.

For the meta-analysis, we used tau-square to represent heterogeneity. Funnel plots and Egger’s test were used to determine potential publication bias even when fewer than 10 studies were included. If publication bias was statistically significant, we further applied trim-and-fill approaches to potentially impute missing studies.^23^^,^^24^ For the dose-response meta-analysis, we employed the variance partition coefficient—an extension of the I-square statistic in the one-stage dose-response meta-analysis—to evaluate heterogeneity. All analyses were conducted using Comprehensive Meta-Analysis (version 4) for meta-analysis and R software (dosresmeta package version 2.0.1) for dose-response meta-analysis. Statistical significance was set at a two-tailed *p*-value of less than 0.05.

### Role of the funding source

The funding source had no role in the study design, collection, analysis, interpretation of data, writing of the manuscript, and decision to submit the paper for publication. All authors contributed important intellectual content during manuscript revision, had full access to all the data in the study, and accepted responsibility to submit for publication.

## Results

**Fig. 1:**
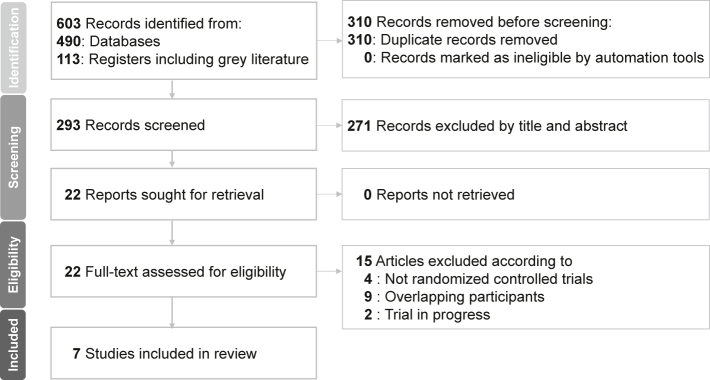
**Flowchart of studyselection.**

The PRISMA flowchart is presented in Fig. 1. We identified 603 studies from searches of databases and registries. After duplicate removal and screening of titles and abstracts, 22 studies remained. Following full-text assessment, 15 studies were excluded for various reasons (eTable 3), and seven were included in our final analysis. Table 1 and eTable 4 summarise the primary characteristics of the included studies. The seven eligible studies encompassed 1789 participants,^11^, ^12^, ^13^, ^14^, ^15^^,^^25^ (NLM, NCT003771664) with a mean age of 38.7 ± 12.2 (standard deviation) years, of whom 72% (n = 1294) were female. All studies included a population with MDD, and two studies^11^^,^^25^ specified the time from onset to the postpartum period. The treatment duration in each study was 14 days, with doses ranging from 20 to 50 mg per day. All included studies used the HAMD-17^11^, ^12^, ^13^, ^14^, ^15^^,^^25^ (NLM, NCT003771664) to assess depression severity, and five also employed the MADRS^11^^,^^13^, ^14^, ^15^^,^^25^ for assessing depression severity. Moreover, for anxiety assessment, five studies^11^^,^^13^, ^14^, ^15^^,^^25^ used the HAM-A, and one study^15^ used a subscale of the HAMD-17. Additionally, there were no head-to-head trials directly comparing zuranolone with other antidepressants.

**Table 1:** Characteristics of included studies.

Study	Study design	Diagnosis (diagnostic criteria)	Number of cases (female/male)	Age, year (SD)	Country	Race (%)^a^
NCT03771664	Double-blind, parallel	MDD (DSM-5)	Placebo: 43 (31/12) Zuranolone 30 mg: 43 (28/15)	42.0 (11.6) 44.6 (12.5)	USA	White (70.9) African American (26.7) Other (2.4)
Gunduz-Bruce et al.^14^	Double-blind, parallel	MDD (DSM-5)	Placebo: 44 (30/14) Zuranolone 30 mg: 45 (25/20)	38.3 (12.2) 49.1 (13.6)	USA	White (25.8) African American (71.9) Other (2.2)
Deligiannidis et al.^11^	Double-blind, parallel	Postpartum-onset MDD (DSM-5)	Placebo: 74 (74/0) Zuranolone 30 mg: 76 (76/0)	27.4 (5.3) 29.3 (5.4)	USA	White (56.0) African American (41.3) Other (2.7)
Kato et al.^12^	Double-blind, parallel	MDD (DSM-5)	Placebo: 82 (47/35) Zuranolone 20 mg: 85 (49/36) Zuranolone 30 mg: 82 (47/35)	40.8 (10.6) 39.3 (12.6) 38.8 (12.0)	Japan	Asian (100.0)
Clayton et al.^15^	Double-blind, parallel	MDD (DSM-5)	Placebo: 157 (106/51) Zuranolone 20 mg: 159 (112/47) Zuranolone 30 mg: 166 (121/45)	41.4 (12.2) 41.9 (12.2) 42.3 (11.8)	USA	White (60.0) African American (36.1) Other (3.9)
Clayton et al.^13^	Double-blind, parallel	MDD (DSM-5)	Placebo: 269 (166/103) Zuranolone 50 mg: 268 (186/82)	40.1 (12.6) 39.4 (12.3)	USA	White (69.8) African American (22.5) Other (7.6)
Deligiannidis^25^^,^^26^	Double-blind, parallel	Postpartum-onset MDD (DSM-5)	Placebo: 98 (98/0) Zuranolone 50 mg: 98 (98/0)	31.0 (6.0) 30.0 (5.9)	USA, Spain, UK	White (69.9) African American (21.9) Other (8.2)

Abbreviations: DSM-5: Diagnostic and Statistical Manual of Mental Disorders, Fifth Edition; MDD: major depressive disorder; SD: standard deviation.

a Other included Asian, American Indian, Alaska Native, Native Hawaiian, Pacific Islander, multiple, other race, and/or not reported.

**Fig. 2:**
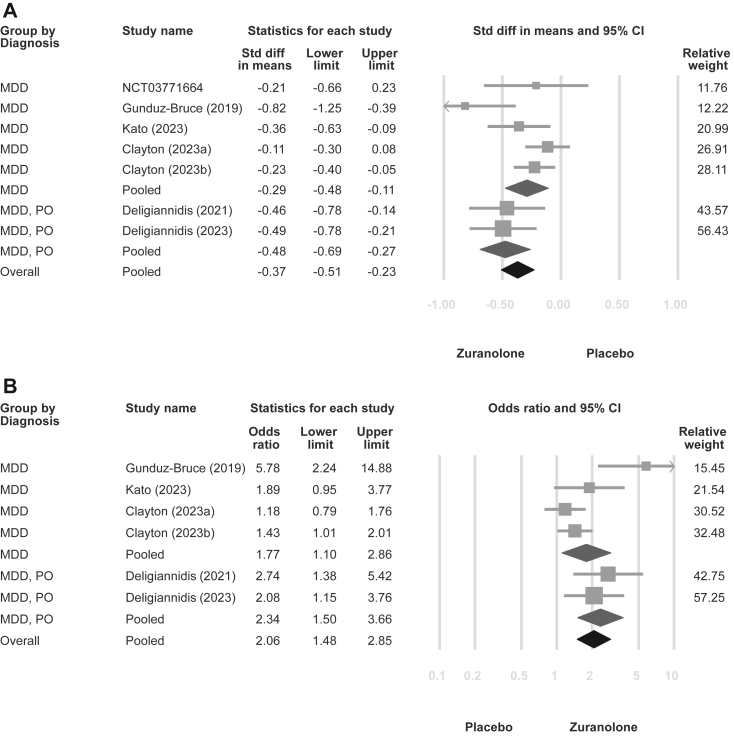

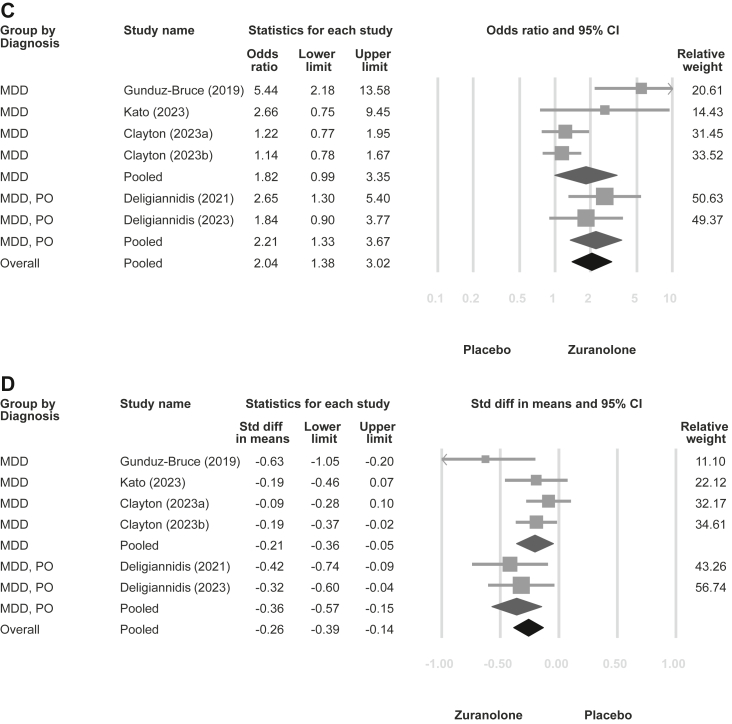

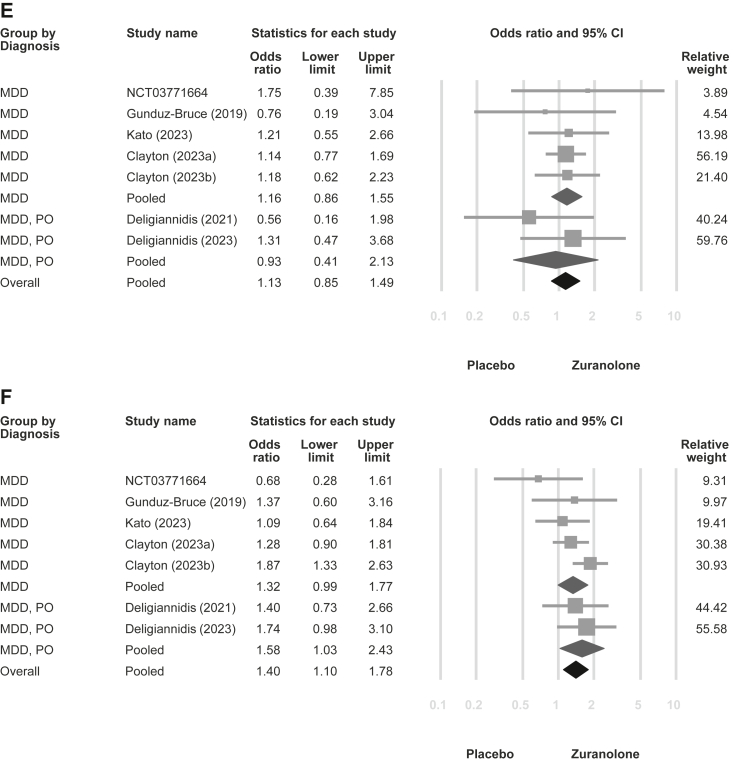
**Forest plot of zuranolone vs. placebo for major depressive disorder with subgroup analyses stratified by general major depressive disorder and postpartum-onset major depressive disorder.** (A) Depression symptoms; (B) Response rate of depression; (C) Remission rate of depression; (D) Anxiety symptoms; (E) Overall dropout rate; (F) Any side effects rate. Abbreviations: MDD: major depressive disorder; PO: postpartum-onset.

In the meta-analysis, zuranolone reduced depressive symptoms (SMD = −0.37, 95% CIs = −0.51 to −0.23, tau-square = 0.021; Fig. 2A) and exhibited higher response (OR = 2.06; 95% CIs = 1.48–2.85, tau-square = 0.121; Fig. 2B) and remission rates (OR = 2.04; 95% CIs = 1.38–3.02, tau-square = 0.179; Fig. 2C) than the placebo. Furthermore, zuranolone alleviated anxiety symptoms (SMD = −0.26, 95% CIs = −0.39 to −0.14, tau-square = 0.008; Fig. 2D). There was no significant difference in dropout rate between the treatment and placebo groups (OR = 1.13, 95% CIs = 0.85–1.49, tau-square < 0.001; Fig. 2E). Side effects were more frequently observed in the zuranolone group than in the placebo group (OR = 1.40, 95% CIs = 1.10–1.78, tau-square = 0.013; Fig. 2F). Furthermore, as all the included studies employed the HAMD-17 scale for evaluating depressive symptoms, the sensitivity analysis for this outcome is illustrated in eFigure 1 (MD = −2.84, 95% CIs = −3.92 to −1.76, tau-square = 1.498). The leave-one-out sensitivity analysis for all outcomes showed similar findings (eFigure 2).

Fig. 2 illustrates the subgroup analysis categorised by postpartum-onset MDD vs. general MDD in terms of depressive symptoms (postpartum-onset MDD vs. general MDD: SMD = −0.48 vs. −0.29; *p* = 0.198), response rate (OR = 2.34 vs. 1.77; *p* = 0.406), remission rate (OR = 2.21 vs. 1.82; *p* = 0.628), anxiety symptoms (SMD = −0.36 vs. −0.21; *p* = 0.249), dropout rate (OR = 0.93 vs. 1.16; *p* = 0.606), and side effects rate (OR = 1.58 vs. 1.32; *p* = 0.502). The subgroup analysis did not show significant differences in outcomes between these two categories. Additionally, possible confounding factors, including initial depression severity, age, and sex of the participants, were used for the meta-regression, although none of these factors were significant (eTable 5).

**Fig. 3:**
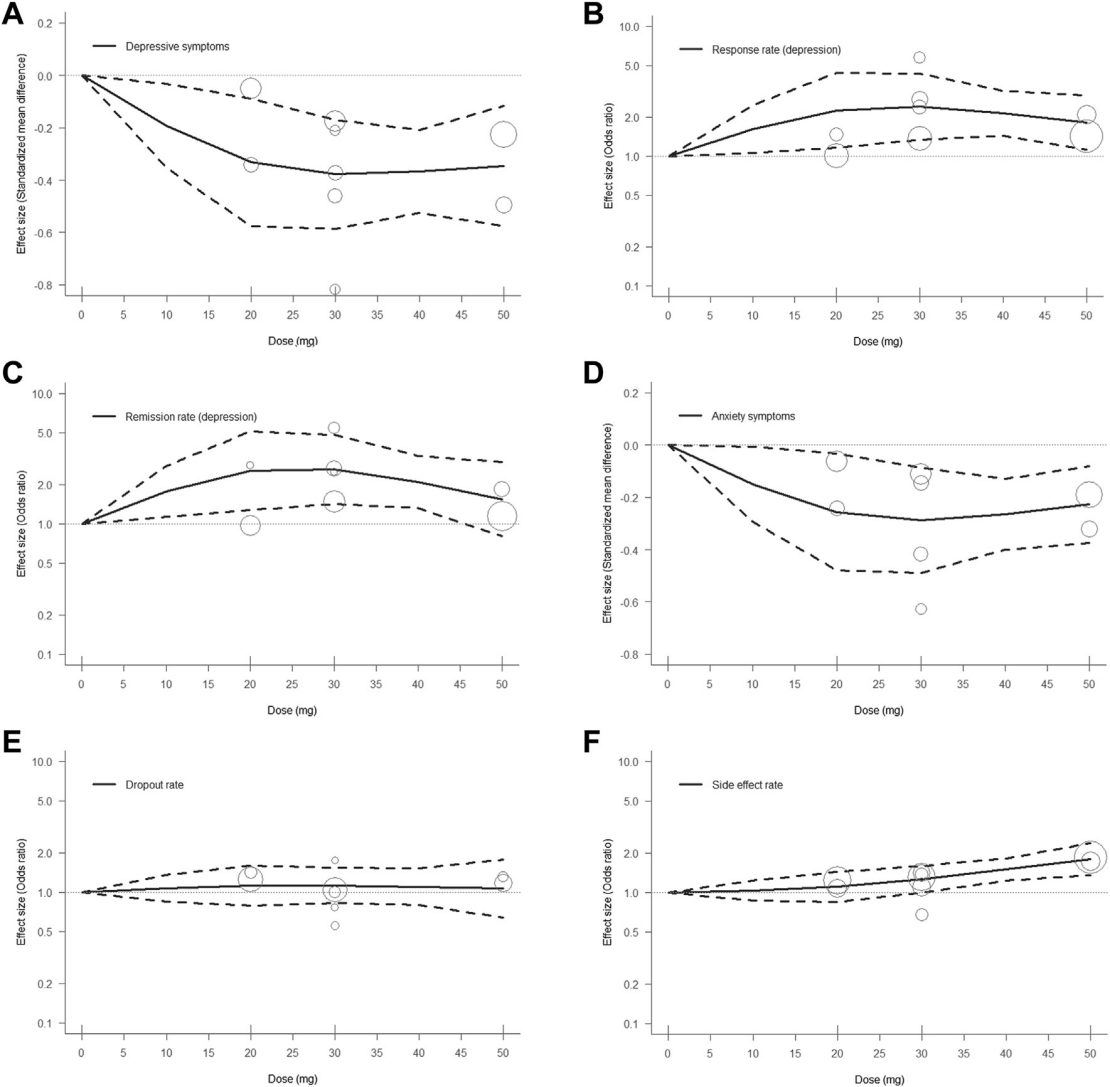
**Dose-response relationship between daily zuranolone doses and effect sizes in major depressive disorder.** (A) Depression symptoms; (B) Response rate of depression; (C) Remission rate of depression; (D) Anxiety symptoms; (E) Overall dropout rate; (F) Any side effects rate. Solid line: pooled point estimates; dotted line: 95% confidence interval; short vertical lines on the x-axis: zuranolone dose of included studies; open circles: outcome markers for all included studies, whose size represents the reciprocal of the standard error of the effect size.

In the dose-response meta-analysis, the curve indicated a decrease in depression severity with an increase in dose (Fig. 3A). Upon reaching a daily dose of 30 mg of zuranolone, the curve depicting the SMD value reached its lowest point (SMD = −0.38, 95% CIs = −0.58 to −0.17; Table 2). Subsequently, the curve levelled off on increasing the dose to 50 mg, and the SMD value remained relatively stable (SMD = −0.35, 95% CIs = −0.58 to −0.12). Similar trends were observed for response and remission rates, with curves showing enhanced efficacy (OR values) with increasing doses (Fig. 3B and C). The peak efficacy was observed at a dosage of 30 mg (response rate: OR = 2.41, 95% CIs = 1.33–4.35; remission rate: OR = 2.61, 95% CIs = 1.41–4.82; Table 2), after which the curve relatively flattened. Similarly, the dose-response relationship of zuranolone for anxiety symptoms was similar to that for depressive symptoms, with the effects plateauing at a dose of 30 mg zuranolone (SMD = −0.29, 95% CIs = −0.49 to −0.08; Table 2 and Fig. 3D). Moreover, there was no significant difference in dropout rate between placebo and various zuranolone doses (Fig. 3E). However, the incidence of side effects increased with increasing dose (30 mg: OR = 1.26, 95% CIs = 1.00–1.59; 50 mg: OR = 1.79, 95% CIs = 1.35–2.39; Table 2 and Fig. 3F). Furthermore, the sensitivity analysis at different fixed percentiles showed similar findings (eFigure 3).

**Table 2:** Estimated effect sizes from dose-response meta-analysis.

Outcome	Zuranolone dose				
	10 mg	20 mg	30 mg	40 mg	50 mg
Depressive symptoms, SMD	−0.19 (−0.35, −0.03)∗	−0.33 (−0.58, −0.09)∗	−0.38 (−0.58, −0.17)∗	−0.37 (−0.53, −0.21)∗	−0.35 (−0.58, −0.12)∗
Response rate (depression), OR	1.62 (1.06, 2.48)∗	2.25 (1.16, 4.36)∗	2.41 (1.33, 4.35)∗	2.15 (1.45, 3.20)∗	1.82 (1.13, 2.93)∗
Remission rate (depression), OR	1.77 (1.13, 2.79)∗	2.56 (1.28, 5.14)∗	2.61 (1.41, 4.82)∗	2.09 (1.32, 3.30)∗	1.55 (0.81, 2.98)
Anxiety symptoms, SMD	−0.15 (−0.29, −0.01)∗	−0.26 (−0.48, −0.03)∗	−0.29 (−0.49, −0.08)∗	−0.26 (−0.40, −0.13)∗	−0.23 (−0.37, −0.08)∗
Dropout rate, OR	1.08 (0.85, 1.36)	1.13 (0.79, 1.60)	1.13 (0.83, 1.54)	1.10 (0.79, 1.52)	1.07 (0.64, 1.77)
Side effect rate, OR	1.03 (0.87, 1.23)	1.11 (0.85, 1.44)	1.26 (1.00, 1.59)∗	1.50 (1.23, 1.83)∗	1.79 (1.35, 2.39)∗

Abbreviations: OR: odds ratio; SMD: standardized mean difference.

An asterisks indicates statistical significance.

Detailed quality assessments using the Cochrane Risk of Bias 2 tool are documented in eTable 6 and eFigure 4. All included trials were judged to have a low risk of bias. For the meta-analysis, the funnel plots are depicted in eFigure 5. Egger's test results for depressive response rate, remission rate, and anxiety symptoms were significant, suggesting the possibility of publication bias. The adjusted funnel plots obtained using the trim-and-fill method are presented in eFigure 6. For the dose-response meta-analysis, the variance partition coefficient is shown in eFigure 7.

## Discussion

From a meta-analysis perspective, zuranolone afforded better efficacy than a placebo in reducing depressive symptoms, depressive response and remission rates, and anxiety symptoms. Regarding safety, although the groups did not differ in terms of dropout rate, the zuranolone group had a higher rate of side effects than the placebo group. Considering the dose in a dose-response meta-analysis, zuranolone at a daily dose of 30 mg exerted the most effective response compared with the placebo. Increasing the dose beyond this point (30 mg–50 mg) does not seem to enhance the effect and instead increases the incidence of side effects. Moreover, the subgroup analysis did not demonstrate a difference in zuranolone efficacy and safety between postpartum-onset MDD and general MDD.

Since the late 1980s, the dominant paradigm for treating depression has been largely guided by the monoaminergic hypothesis. Our findings suggest that zuranolone has the potential to transform antidepressant drug development. Recent studies have highlighted the relationship between GABA levels and depression. Acute and chronic stress^27^ lower GABA levels in the frontal and prefrontal cortices, consequently terminating the inhibition of the hypothalamus-pituitary-adrenal axis,^28^ leading to a major deficit in stress response inhibition. Low GABA levels in unmedicated patients with depression were found in the prefrontal, occipital, and anterior cingulate cortices,^29^ as observed by positron emission tomography imaging. Zuranolone, as a GABA_A_R-positive allosteric modulator binding to α or β subunits, leads to enhanced phasic (synaptic GABA_A_R) and tonic (extrasynaptic GAB_A_AR) inhibitory actions. This effect helps maintain a balanced brain network (e.g., the central executive network, default mode network, and salience network), allowing the brain to respond appropriately to harmful stimuli.^30^ Moreover, zuranolone can effectively alleviate anxiety. Allopregnanolone modulates innate responses to anxiogenic stimuli in the central nervous system and anxiety responses by interacting with GABA_A_ and N-methyl-d-aspartate glutamate receptors.^31^ Anxiety modulation has been associated with the interference of neural activity in the amygdala by metabolites of progesterone and progesterone itself.^32^ Low serum neurosteroid levels have been observed in patients with generalised anxiety disorder and social phobia.^33^ Furthermore, progesterone administration has been associated with a sedative-anxiolytic effect in humans.^34^ In conclusion, our meta-analysis revealed that zuranolone could effectively improve depression and anxiety, supporting previous theoretical foundations.

Based on the current meta-analysis, increasing the zuranolone dose increased the incidence of adverse events when compared with those of the placebo (OR = 1.40). This can be explained by the results of another clinical trial, which focused on the hypnotic effects of zuranolone and aimed to manage the frequently coexisting symptoms of insomnia observed in a population with postpartum depression.^26^ Common side effects, such as somnolence, dizziness, sedation, and headache, were noted in the included studies. Allopregnanolone may shorten non-rapid eye movement sleep latency and play a role in physiological sleep,^35^ potentially linked to the observed adverse effects. Moreover, a document published by the US FDA = noted that in a study of general MDD,^13^ participants randomised to zuranolone (1%) experienced suicidal ideation or behaviour more frequently than participants in the placebo group (0%).^36^ This imbalance has not been observed in studies of postpartum-onset MDD. This difference may have also contributed to the FDA's decision to prioritise approving zuranolone for the treatment of postpartum-onset MDD rather than general MDD. Despite the aforementioned adverse events, the comparable dropout rates between the placebo and zuranolone groups suggested that zuranolone was generally well tolerated.

Although subgroup analysis failed to detect differences in zuranolone efficacy between postpartum-onset and general MDD, pooled point estimates for depression and anxiety revealed better outcomes in postpartum depression than those in general MDD. Zuranolone, an allopregnanolone derivative, could be beneficial in postpartum depression and MDD,^37^ given that decreased allopregnanolone levels have been documented in individuals with these conditions. The antidepressant characteristics of zuranolone are associated with its role as a positive allosteric modulator of the GABA_A_ receptor. Our subgroup analysis results may be attributed to the following reasons: (1) the inclusion criteria for unspecified-onset MDD may have unintentionally included participants with postpartum-onset depression, which could have mixed the observed effect with that of postpartum depression; (2) the previously observed association between decreased allopregnanolone levels and MDD in the general population^37^ may be equally important in the context of postpartum-onset MDD; and (3) the limited sample size in the trial may have led to underpowered statistical power. Accordingly, the differences between the two subgroups may not be as pronounced as expected. Moreover, a closer look at subgroup analysis revealed potential heterogeneity that may arise from studies targeting general MDD (Fig. 2A). Effect sizes were found to be nearly identical in two studies investigating postpartum-onset MDD. However, of the five studies on general MDD, with the exception of the initial study by Gunduz-Bruce et al.,^14^ which showed the best effect, the subsequent four studies did not appear to replicate comparable effect sizes. Notably, two of these four studies showed no difference in the treatment effect of zuranolone compared with placebo (NLM, NCT03771664 and Clayton et al.^15^). Therefore, this might imply that zuranolone potentially exhibits a more consistent therapeutic impact on postpartum-onset MDD compared to general MDD. General MDD may cover a wider range of MDD subtypes and exhibit considerable heterogeneity.^38^ Finally, our meta-regression analysis did not detect any confounding effects of variables such as depression severity, sex, or age on our results.

Our dose-response meta-analysis indicated that the efficacy of zuranolone increased with daily dosage. However, a ceiling effect was apparent at a daily dose of 30 mg for depression reduction and anxiety relief. It has been reported that the sigmoidal dose-response curve of steroids might originate from the conditional fulfilment of Michaelis–Menten kinetics.^39^ Further exploration is needed to determine whether this is due to a saturable allosteric site in GABA_A_ receptors. However, the incidence of side effects was positively related to dose, with the same trend for daily doses above 30 mg. Thus, considering both efficacy and safety, 30 mg of zuranolone may be a suitable approach for patients with MDD. This dosage could deliver desirable effects in anxiety alleviation and improvement of response and remission rates while diminishing the risk of any side effects.

To the best of our knowledge, this is the first meta-analysis to discuss the efficacy and safety of zuranolone in the treatment of MDD. A dose-response meta-analysis further explored the relationship between the dose of zuranolone and treatment outcomes in patients with MDD. However, the limitations of the current study need to be addressed. First, publication bias was observed in the results of depression response, depression remission, and improvement in anxiety symptoms, as indicated by publication bias in the corresponding funnel plots and Egger’s tests (eFigure 5B–D). We attempted to confront publication bias by searching for grey literature and unpublished data and applying the trim-and-fill method to account for the impact of potentially missing studies (eFigure 6A–C). Moreover, we utilised the leave-one-out analysis to potentially pinpoint the source of heterogeneity (eFigure 2), which appears to be attributable to the Gunduz-Bruce et al. study^14^ because of its large deviation from the main results. In summary, although the conclusions highlight the efficacy of zuranolone, the findings suggest that results in these areas may be overestimated and should therefore be interpreted cautiously. Second, all studies included in our meta-analysis, except for Clayton et al.^15^ with a 6-month follow-up period, focused primarily on the immediate effect immediately after the 14-day regimen. Thus, our outcome could only represent the short-term antidepressant efficacy of zuranolone, and the duration remains unknown and requires further investigation in future trials. Third, currently published RCTs are limited to comparisons between zuranolone and placebo. Studies comparing the therapeutic effects of zuranolone with those of other antidepressants are still lacking in the literature; therefore, the relative effectiveness of zuranolone when compared with that of other antidepressants is yet to be established. Subsequent research efforts should be oriented toward addressing this point.

Our meta-analysis demonstrated that zuranolone could alleviate depressive symptoms, increases response and remission rates, and reduce concurrent anxiety symptoms in MDD patients. Although safety issues seem to be addressed, with dropout rates comparable to those of the placebo, it is crucial to remain vigilant regarding potential side effects. Considering the dose-response relationship of efficacy and safety trends, a daily dose of 30 mg of zuranolone emerged as a favourable option, reducing depression and anxiety symptoms. Despite the associated side effects, the dropout rate is comparable to that of the placebo. Further research is required to validate whether zuranolone is more effective against postpartum depression than against general MDD.

## Contributors

CWH conceived the research idea for the study. CWH led the study design with YWL. YWL and CWH were responsible for data acquisition and extraction, and subsequent verification of the data. CWH performed statistical analysis. YWL drafted the manuscript first, and then YKT, KCH, CSL, PTT, PYL, CCL, and CWH revised the manuscript. All authors contributed important intellectual content during manuscript revision, had full access to all the data in the study, and accept responsibility to submit for publication.

## Data sharing statement

The data that support the findings of this study are available from the corresponding author, CWH, upon reasonable request.

## Declaration of interests

The authors report no financial interests or potential conflicts of interest.
