# Supplementary material for: Efficacy and safety of zuranolone in major depressive disorder: a meta-analysis of factor effect and dose-response analyses
Source: eClinicalMedicine. 2023 Nov 16;66:102308. doi: 10.1016/j.eclinm.2023.102308 (PMC10690540; doi:10.1016/j.eclinm.2023.102308)
Supplement: Supplemental Data [file mmc1.pdf]

Supplemental data

Efficacy and safety of zuranolone in major depressive disorder: a meta-analysis of factor effect and dose-response analyses

Running Title: zuranolone for MDD

Yu-Wei Lin[1], Yu-Kang Tu[2,3], Kuo-Chuan Hung[4], Chih-Sung Liang[5,6], Ping-Tao Tseng[7,8,9,10], Pao-Yen Lin[1,11], Edward Chia-Cheng Lai[12], Chih-Wei Hsu[1,*]

[1] Department of Psychiatry, Kaohsiung Chang Gung Memorial Hospital and Chang Gung University College of Medicine, Kaohsiung, Taiwan
[2] Institute of Epidemiology and Preventive Medicine, National Taiwan University College of Public Health, Taipei, Taiwan
[3] Department of Dentistry, National Taiwan University Hospital, Taipei, Taiwan
[4] Department of Anesthesiology, Chi Mei Medical Center, Tainan, Taiwan
[5] Department of Psychiatry, Beitou Branch, Tri-Service General Hospital, National Defense Medical Center, Taipei, Taiwan
[6] Department of Psychiatry, National Defense Medical Center, Taipei, Taiwan
[7] Prospect Clinic for Otorhinolaryngology & Neurology, Kaohsiung, Taiwan
[8] Institute of Biomedical Sciences, National Sun Yat-sen University, Kaohsiung, Taiwan
[9] Department of Psychology, College of Medical and Health Science, Asia University, Taichung, Taiwan
[10] Institute of Precision Medicine, National Sun Yat-sen University, Kaohsiung City, Taiwan
[11] Institute for Translational Research in Biomedical Sciences, Kaohsiung Chang Gung Memorial Hospital, Kaohsiung, Taiwan
[12] School of Pharmacy, Institute of Clinical Pharmacy and Pharmaceutical Sciences, College of Medicine, National Cheng Kung University, Tainan, Taiwan

*** Corresponding author:**
Chih-Wei Hsu, MD
Department of Psychiatry, Kaohsiung Chang Gung Memorial Hospital
No. 123, Dapi Road, Niaosong District, Kaohsiung City 833, Taiwan
Telephone number: 886-7-7317123 ext. 8753
Fax number: 886-7-7326817
E-mail address: harwicacademia@gmail.com

**Content**	**Page**
eTable 1. PRISMA Checklist	1-2
eTable 2. Detailed search strategy	3
eTable 3. Excluded studies and reason	4-5
eTable 4. Supplementary characteristics of included studies	6
eTable 5. Meta-regression for examining moderator relationships	7
eTable 6. Detailed quality assessment of included studies using Cochrane risk of bias 2 tool	8
eFigure 1. Sensitivity analysis of forest plot for depressive symptom outcome	9
eFigure 2. Sensitivity analysis of forest plot using the leave-one-out method	10-12
eFigure 3. Sensitivity analysis at fixed percentiles (10%, 75%, 95%)	13-15
eFigure 4. Summary of quality assessment of included studies using Cochrane risk of bias 2 tool	16
eFigure 5. Funnel plots with Egger's test	17-19
eFigure 6. Funnel plots after trim-and-fill method	20-21
eFigure 7. Variation partition coefficients	22-24
References	25

eTable 1. PRISMA Checklist

Section and Topic	#	Checklist item	Location
**TITLE**			
Title	1	Identify the report as a systematic review.	Title page
**ABSTRACT**			
Abstract	2	See the PRISMA 2020 for Abstracts checklist.	Summary
**INTRODUCTION**			
Rationale	3	Describe the rationale for the review in the context of existing knowledge.	1-2
Objectives	4	Provide an explicit statement of the objective(s) or question(s) the review addresses.	1-2
**METHODS**			
Eligibility criteria	5	Specify the inclusion and exclusion criteria for the review and how studies were grouped for the syntheses.	3
Information sources	6	Specify all databases, registers, websites, organisations, reference lists and other sources searched or consulted to identify studies. Specify the date when each source was last searched or consulted.	3
Search strategy	7	Present the full search strategies for all databases, registers and websites, including any filters and limits used.	3, eTable 2
Selection process	8	Specify the methods used to decide whether a study met the inclusion criteria of the review, including how many reviewers screened each record and each report retrieved, whether they worked independently, and if applicable, details of automation tools used in the process.	3-4
Data collection process	9	Specify the methods used to collect data from reports, including how many reviewers collected data from each report, whether they worked independently, any processes for obtaining or confirming data from study investigators, and if applicable, details of automation tools used in the process.	4
Data items	10a	List and define all outcomes for which data were sought. Specify whether all results that were compatible with each outcome domain in each study were sought (e.g., for all measures, time points, analyses), and if not, the methods used to decide which results to collect.	4
	10b	List and define all other variables for which data were sought (e.g., participant and intervention characteristics, funding sources). Describe any assumptions made about any missing or unclear information.	4
Study risk of bias assessment	11	Specify the methods used to assess risk of bias in the included studies, including details of the tool(s) used, how many reviewers assessed each study and whether they worked independently, and if applicable, details of automation tools used in the process.	4-5
Effect measures	12	Specify for each outcome the effect measure(s) (e.g., risk ratio, mean difference) used in the synthesis or presentation of results.	4-5
Synthesis methods	13a	Describe the processes used to decide which studies were eligible for each synthesis (e.g., tabulating the study intervention characteristics and comparing against the planned groups for each synthesis (item #5)).	4-5
	13b	Describe any methods required to prepare the data for presentation or synthesis, such as handling of missing summary statistics, or data conversions.	4-5
	13c	Describe any methods used to tabulate or visually display results of individual studies and syntheses.	5
	13d	Describe any methods used to synthesize results and provide a rationale for the choice(s). If meta-analysis was performed, describe the model(s), method(s) to identify the presence and extent of statistical heterogeneity, and software package(s) used.	5-6
	13e	Describe any methods used to explore possible causes of heterogeneity among study results (e.g., subgroup analysis, meta-regression).	5
	13f	Describe any sensitivity analyses conducted to assess robustness of the synthesized results.	5
Reporting bias assessment	14	Describe any methods used to assess risk of bias due to missing results in a synthesis (arising from reporting biases).	5
Certainty assessment	15	Describe any methods used to assess certainty (or confidence) in the body of evidence for an outcome.	NA
**RESULTS**			
Study selection	16a	Describe the results of the search and selection process, from the number of records identified in the search to the number of studies included in the review, ideally using a flow diagram.	7, Figure 1
	16b	Cite studies that might appear to meet the inclusion criteria, but which were excluded, and explain why they were excluded.	eTable 3
Study characteristics	17	Cite each included study and present its characteristics.	7, Table 1, eTable 4
Risk of bias	18	Present assessments of risk of bias for each included study.	eTable 6, eFigure 4
Results of individual studies	19	For all outcomes, present, for each study: (a) summary statistics for each group (where appropriate) and (b) an effect estimates and its precision (e.g., confidence/credible interval), ideally using structured tables or plots.	7-8, Figure 2-3
Results of syntheses	20a	For each synthesis, briefly summarise the characteristics and risk of bias among contributing studies.	7, eFigure 4-7, eTable 6

	20b	Present results of all statistical syntheses conducted. If meta-analysis was done, present for each the summary estimate and its precision (e.g., confidence/credible interval) and measures of statistical heterogeneity. If comparing groups, describe the direction of the effect.	7-8, Figure 2-3
	20c	Present results of all investigations of possible causes of heterogeneity among study results.	7-8, eFigure 1-3
	20d	Present results of all sensitivity analyses conducted to assess the robustness of the synthesized results.	7-8, eFigure 1-3
Reporting biases	21	Present assessments of risk of bias due to missing results (arising from reporting biases) for each synthesis assessed.	8, eFigure 5-6
Certainty of evidence	22	Present assessments of certainty (or confidence) in the body of evidence for each outcome assessed.	NA
**DISCUSSION**			
Discussion	23a	Provide a general interpretation of the results in the context of other evidence.	9
	23b	Discuss any limitations of the evidence included in the review.	11-12
	23c	Discuss any limitations of the review processes used.	11
	23d	Discuss implications of the results for practice, policy, and future research.	12-13
**OTHER INFORMATION**			
Registration and protocol	24a	Provide registration information for the review, including register name and registration number, or state that the review was not registered.	3
	24b	Indicate where the review protocol can be accessed, or state that a protocol was not prepared.	3
	24c	Describe and explain any amendments to information provided at registration or in the protocol.	3
Support	25	Describe sources of financial or non-financial support for the review, and the role of the funders or sponsors in the review.	14
Competing interests	26	Declare any competing interests of review authors.	14
Availability of data, code and other materials	27	Report which of the following are publicly available and where they can be found: template data collection forms; data extracted from included studies; data used for all analyses; analytic code; any other materials used in the review.	14

eTable 2. Keywords and search results in different databases

Database	Keyword	Filter	Date	Results
PubMed	(depress* OR 'affective' OR 'mood') AND ('zuranolone' OR 'SAGE-217' OR 'S-812217')	Not applied	August 20, 2023	50
Embase	(depress* OR 'affective' OR 'mood') AND ('zuranolone' OR 'SAGE-217' OR 'S-812217')	Title Abstract Keyword	August 20, 2023	110
Cochrane CENTRAL	(depress* OR 'affective' OR 'mood') AND ('zuranolone' OR 'SAGE-217' OR 'S-812217')	Title Abstract Keyword	August 20, 2023	78
Web of Science	(depress* OR 'affective' OR 'mood') AND ('zuranolone' OR 'SAGE-217' OR 'S-812217')	Not applied	August 20, 2023	82
ProQuest	(depress* OR 'affective' OR 'mood') AND ('zuranolone' OR 'SAGE-217' OR 'S-812217')	Scholarly Journal	August 20, 2023	191
ScienceDirect	('depress' OR 'affective' OR 'mood') AND ('zuranolone' OR 'SAGE-217' OR 'S-812217')	Title Abstract Keyword	August 20, 2023	21
Clinical Key	(depress* OR 'affective' OR 'mood') AND ('zuranolone' OR 'SAGE-217' OR 'S-812217')	Journal/Randomized Controlled Trials	August 20, 2023	36
ClinicalTrials.gov	(depress* OR 'affective' OR 'mood') AND ('zuranolone' OR 'SAGE-217' OR 'S-812217')	Condition or disease Intervention/ Treatment	August 20, 2023	11

Keyword adjusted as below in ScienceDirect due to wildcard* was not applicable: (‘depress’ OR 'affective' OR 'mood') AND ('zuranolone' OR 'SAGE-217' OR 'S-812217'); Cochrane CENTRAL and ClinicalTrials.gov were classified as registries in the PRISMA flowchart (Figure 1).

Grey literature N = 24

Use the keywords "zuranolone" OR "SAGE-217 " OR "S-812217," to search the following grey literature.

1. Airiti Library (https://www.airitilibrary.com/Home/Index), n = 0
2. CADTH checklist (https://www.cadth.ca/), n = 0
3. ISRCTN Registry (https://www.isrctn.com/), n = 0
4. OAIster (https://oaister.worldcat.org/), n = 3
5. World Health Organization International Clinical Trials Registry Platform (ICTRP) (https://trialsearch.who.int/Default.aspx), n = 21

eTable 3. Excluded studies and reasons

Reasons	Citations
**Not randomized controlled trials**	
**Open label trial**	National Library of Medicine (U.S.). (2018, Aug. – 2019, May). A Study to Evaluate SAGE-217 in Participants With Bipolar I/II Disorder With a Current Major Depressive Episode. Identifier: NCT03692910. https://clinicaltrials.gov/study/NCT03692910
**Open label trial**	National Library of Medicine (U.S.). (2019, Mar. -). A Study to Evaluate SAGE-217 in Adult Participants With Major Depressive Disorder (MDD). Identifier: NCT03864614. https://www.clinicaltrials.gov/study/NCT03864614
**Open label trial**	Cutler, A., Aaronson, S., Mattingly, G., Wilkinson, S., Lasser, R., Nandy, I., ... & Kanes, S. (2021). P. 0672 Zuranolone 30 mg in major depressive disorder: results through 1-year follow-up from the Phase 3, open-label, SHORELINE study. European *Neuropsychopharmacology, 53*, S492-S493.
**Open label trial**	Cutler, A., et al. (2021). "Zuranolone in the treatment of major depressive disorder in patients ≥65 years of age: Outcomes from the phase 3, naturalistic shoreline study." *Neuropsychopharmacology 46*: 214-215.
**Participants overlap with other studies**	
**Gunduz-Bruce et al. (2019)[1]**	Suthoff, E., Kosinski, M., Arnaud, A., Hodgkins, P., Gunduz-Bruce, H., Lasser, R., Silber, C., Sankoh, A. J., Li, H., Werneburg, B., Jonas, J., Doherty, J., Kanes, S. J., & Bonthapally, V. (2022). Patient-reported health-related quality of life from a randomized, placebo-controlled phase 2 trial of zuranolone in adults with major depressive disorder. *Journal of affective disorders*, *308*, 19–26. https://doi.org/10.1016/j.jad.2022.03.068
**Gunduz-Bruce et al. (2019)[1]**	Arnaud, A., Suthoff, E., Stenson, K., Werneburg, B., Hodgkins, P., Bonthapally, V., Jonas, J., Meyer, K., & O'Day, K. (2021). Number Needed to Treat and Number Needed to Harm analysis of the zuranolone phase 2 clinical trial results in major depressive disorder. *Journal of affective disorders*, *285*, 112–119. https://doi.org/10.1016/j.jad.2021.02.027
**Gunduz-Bruce et al. (2019)[1]**	National Library of Medicine (U.S.). (2016, Dec. - 2017, Jan.). A Study to Evaluate SAGE-217 in Participants With Moderate to Severe Major Depressive Disorder. Identifier: NCT03000530. https://clinicaltrials.gov/study/NCT03000530
**Deligiannidis et al. (2021)[2]**	Deligiannidis, K. M., Citrome, L., Huang, M. Y., Acaster, S., Fridman, M., Bonthapally, V., Lasser, R., & Kanes, S. J. (2023). Effect of Zuranolone on Concurrent Anxiety and Insomnia Symptoms in Women With Postpartum Depression. *The Journal of clinical psychiatry*, *84*(1), 22m14475. https://doi.org/10.4088/JCP.22m14475
**Deligiannidis et al. (2021)[2]**	National Library of Medicine (U.S.). (2017, Jan. - 2018, Dec.). A Study to Evaluate SAGE-217 in Participants With Severe Postpartum Depression. Identifier: NCT02978326. https://clinicaltrials.gov/study/NCT02978326

**Kato et al. (2023)**[3]	World Health Organization. (2020, Jul. -). A phase 2 study of S-812217 in patients with major depressive disorder. JPRN-JapicCTI-205276. https://trialsearch.who.int/Trial2.aspx?TrialID=JPRN-JapicCTI-205276
**Clayton et al. (2023a)**[4]	National Library of Medicine (U.S.). (2018, Nov. - 2020, Mar.). A Study to Evaluate the Efficacy of SAGE-217 in the Treatment of Adult Participants With Major Depressive Disorder. Identifier: NCT03672175. https://clinicaltrials.gov/study/NCT03672175
**Clayton et al. (2023b)**[5]	National Library of Medicine (U.S.). (2020, May – 2021, Apr.). A Study to Evaluate the Efficacy of Sage-217 in the Treatment of Adult Participants With Major Depressive Disorder (MDD). Identifier: NCT04442490. https://clinicaltrials.gov/study/NCT04442490
**Deligiannidis et al. (2023)**[6]	National Library of Medicine (U.S.). (2020, Jun. – 2023, Apr.). A Study to Evaluate the Efficacy and Safety of SAGE-217 in Participants With Severe Postpartum Depression (PPD). Identifier: NCT04442503. https://clinicaltrialsgov/show/NCT04442503.
**The trial has not ended**	
**Recruiting**	World Health Organization. (2022, Nov. -). A phase 3 study of S-812217 in combination with an antidepressant in patients with major depressive disorder. JPRN-jRCT2031220423. https://trialsearch.who.int/Trial2.aspx?TrialID=JPRN-jRCT2031220423
**Recruiting**	World Health Organization. (2022, Aug. -). A phase 3 study of S-812217 in patients with major depressive disorder. JPRN-jRCT2031210577. https://trialsearch.who.int/Trial2.aspx?TrialID=JPRN-jRCT2031210577

eTable 4. Supplementary characteristics of included studies

Study	Duration of treatment/follow-up	Average depression severity at baseline/follow-up[1]	Response rate in Depression (%)	Remission rate in Depression (%)	Average anxiety severity at baseline/follow-up[2]	Dropout rate (%)	Any side effects rate (%)
**NCT03771664[7]**	14/72 days	Placebo: -6·4 (change)	Not reported	Not reported	Not reported	7·0	65·1
		Zuranolone 30: -7·9 (change)				11·6	55·8
**Gunduz-Bruce 2019[1]**	14/42 days	Placebo: 25·7/15·4	41·0	26·0	Placebo: -8·60 (change)	11·4	45·5
		Zuranolone 30: 25·2/7·8	79·0	64·0	Zuranolone 30: -13·20 (change)	8·9	53·3
**Deligiannidis 2021[2]**	14/45 days	Placebo: 28·8/15·2	48·0	23·3	Placebo: -12·74 (change)	9·2	52·1
		Zuranolone 30: 28·4/10·6	71·6	44·6	Zuranolone 30: -16·60 (change)	5·2	60·3
**Kato 2023[3]**	14/98 days	Placebo: 24·5/18·3	15·9	3·8	Placebo: 43·8/32·5	12·2	52·4
		Zuranolone 20: 24·8/16·7	22·2	9·9	Zuranolone 20: 45·1/30·3	16·5	54·1
		Zuranolone 30: 24·6/16·3	31·3	8.9	Zuranolone 30: 43·8/30·7	12·2	54·9
**Clayton 2023a[4]**	14/182 days	Placebo: 25·8/14·7	42·6	23·4	Placebo: -8·70 (change)	25·8	52·1
		Zuranolone 20: 25·8/14·3	42·8	23·0	Zuranolone 20: -9·10 (change)	30·3	57·4
		Zuranolone 30: 25·9/13·4	50·3	31·4	Zuranolone 30: -9·40 (change)	26·6	58·9
**Clayton 2023b[5]**	14/42 days	Placebo: 26·9/14·6	47·0	27·1	Placebo: -9·10 (change)	7·1	44·6
		Zuranolone 50: 26·8/12·7	55·9	29·8	Zuranolone 50: -10·4 (change)	8·2	60·1
**Deligiannidis 2023[6]**	14/45 days	Placebo: 24·7/13·1	38·9	16·7	Placebo: 24·7/14·1	7·1	53·1
		Zuranolone 50: 24·4/8·8	57·0	26·9	Zuranolone 50: 24.4/11·6	9·2	66·3

[1] All trials used 17-item Hamilton Depression Rating Scale, and defined the same criteria of response and remission. If the baseline score is not disclosed, the change in score will be shown.

[2] All trials used Hamilton Anxiety Rating Scale except Kato 2023, which use anxiety subscale of 17-item Hamilton Depression Rating Scale. If the baseline score is not disclosed, the change in score will be shown.

eTable 5. Meta-regression for examining moderator relationships

Moderator	Studies (k)	Coefficient with 95% CI	p-Value
Depressive symptoms			
Initial depression severity	6	-0·02 (-0·14,0·10)	0·750
Age, years	7	0·01 (-0·02,0·04)	0·451
Sex, female percentage	7	-0·32 (-1·30,0·65)	0·519
Response rate (depression)			
Initial depression severity	6	0·02 (-0·25,0·29)	0·867
Age, years	6	-0·01 (-0·08,0·05)	0·704
Sex, female percentage	6	0·46 (-1·83,2·74)	0·695
Remission rate (depression)			
Initial depression severity	6	-0·05 (-0·40,0·29)	0·765
Age, years	6	-0·00 (-0·08,0·08)	0·948
Sex, female percentage	6	0·13 (-2·69,2·96)	0·927
Dropout rate			
Initial depression severity	6	-0·04 (-0·30,0·22)	0·750
Age, years	7	0·02 (-0·05,0·09)	0·631
Sex, female percentage	7	-0·53 (-2·94,1·87)	0·664
Side effect rate			
Initial depression severity	6	0·10 (-0·05,0·26)	0·185
Age, years	7	-0·02 (-0·06,0·03)	0·434
Sex, female percentage	7	0·45 (-1·08,1·99)	0·562

eTable 6. Detailed quality assessment of included studies using Cochrane risk of bias 2 tool

Study	Year	Randomization process	Intervention adherence	Missing outcome data	Outcome measurement	Selective reporting	Overall RoB
**NCT03771664[7]**	2018	L	L	L	L	L	L
**Gunduz-Bruce[1]**	2019	L	L	L	L	L	L
**Deligiannidis [2]**	2021	L	L	L[1]	L	L	L
**Kato[3]**	2023	L	L	L[2]	L	L	L
**Clayton[4]**	2023a	L	L	L[3]	L	L	L
**Clayton[5]**	2023b	L	L	L	L	L	L
**Deligiannidis[6]**	2023	L	L	L	L	L	L

[1] The threshold to conduct sensitivity test stated in protocol uploaded at ClinicalTrial.gov was 5%; however, the overall dropout rate was 7.2%, but the threshold was shifted to 10% within method of final article, without sensitivity test presented.

[2] The relatively longer follow-up period of 99 days led to a higher dropout rate of 13.6%. However, if measured the captured timepoint of the 15th day, when the treatment regimen just completed, the dropout rate was 4.0%

[3] The relatively longer follow-up period of 182 days led to a higher dropout rate of 27.02%. However, if measured the captured timepoint of the 15th day, when the treatment regimen just completed, the dropout rate was 6.02%

H, high risk of bias; L, low risk of bias; RoB, risk of bias; S, some concerns.

eFigure 1. Sensitivity analysis of forest plot for depressive symptom outcome

Group by Diagnosis	Study name	Statistics for each study: Difference in means	Lower limit	Upper limit	Relative weight
MDD	NCT03771664	-1.50	-4.64	1.64	12.40
MDD	Gunduz-Bruce (2019)	-7.10	-10.70	-3.50	10.27
MDD	Kato (2023)	-2.00	-3.50	-0.51	25.58
MDD	Clayton (2023a)	-0.91	-2.44	0.62	25.19
MDD	Clayton (2023b)	-1.80	-3.20	-0.40	26.56
MDD	Pooled	-2.14	-3.49	-0.78	
MDD, PO	Deligiannidis (2021)	-4.20	-7.12	-1.28	37.67
MDD, PO	Deligiannidis (2023)	-4.00	-6.27	-1.73	62.33
MDD, PO	Pooled	-4.08	-5.87	-2.28	
Overall	Pooled	-2.84	-3.92	-1.76	

Difference in means and 95% CI

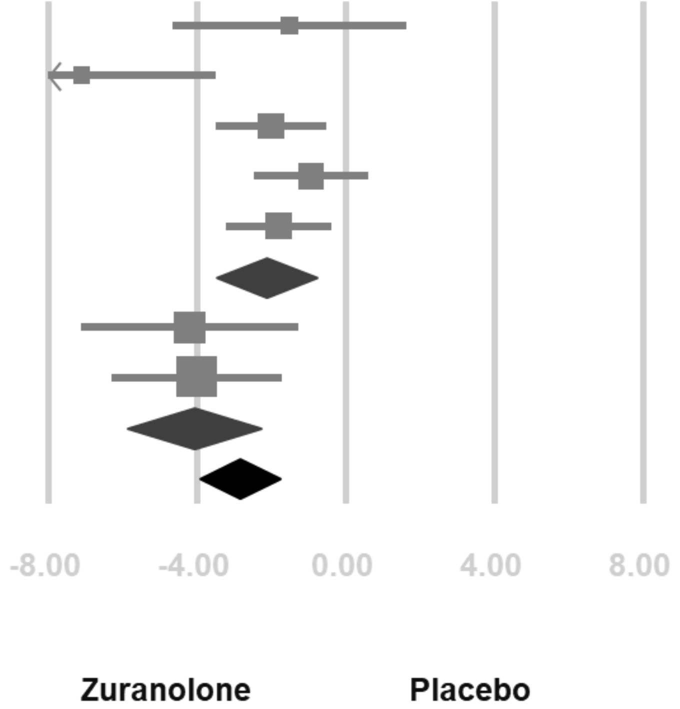

eFigure 2. Sensitivity analysis of forest plot using the leave-one-out method

(A) Depressive symptoms

Study name	Point	Lower limit	Upper limit
NCT03771664	-0.36	-0.53	-0.19
Gunduz-Bruce (2019)	-0.28	-0.41	-0.16
Deligiannidis (2021)	-0.33	-0.50	-0.16
Kato (2023)	-0.35	-0.53	-0.16
Clayton (2023a)	-0.39	-0.55	-0.24
Clayton (2023b)	-0.38	-0.57	-0.19
Deligiannidis (2023)	-0.32	-0.48	-0.15
Pooled	-0.34	-0.50	-0.19

Std diff in means (95%CI) with study removed

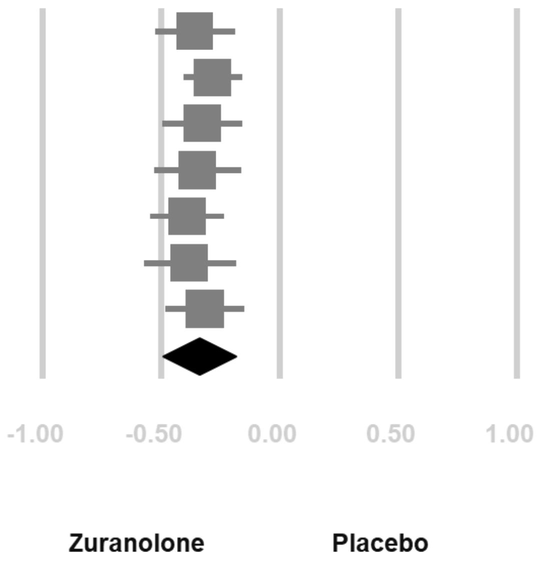

(B) Depression response rate

Study name	Point	Lower limit	Upper limit
Gunduz-Bruce (2019)	1.62	1.23	2.14
Deligiannidis (2021)	1.80	1.21	2.67
Kato (2023)	1.95	1.27	3.01
Clayton (2023a)	2.19	1.45	3.31
Clayton (2023b)	2.15	1.33	3.49
Deligiannidis (2023)	1.92	1.24	2.97
Pooled	1.92	1.33	2.76

Odds ratio (95% CI) with study removed

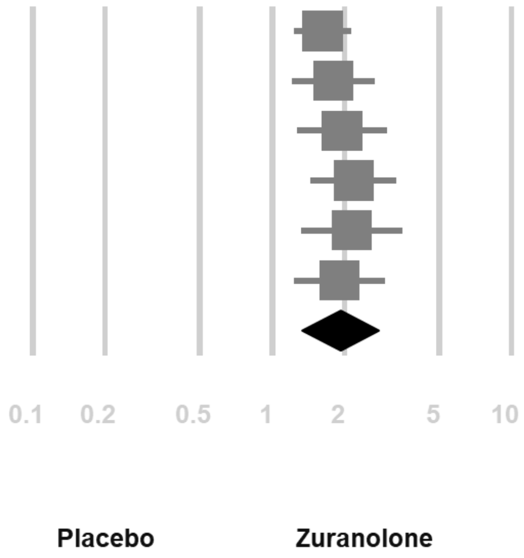

(C) Depression remission rate

Odds ratio (95% CI) with study removed

Study name	Point	Lower limit	Upper limit
Gunduz-Bruce (2019)	1.51	1.09	2.11
Deligiannidis (2021)	1.78	1.09	2.90
Kato (2023)	1.85	1.14	2.99
Clayton (2023a)	2.20	1.24	3.91
Clayton (2023b)	2.23	1.32	3.77
Deligiannidis (2023)	1.95	1.15	3.33
Pooled	1.90	1.22	2.96

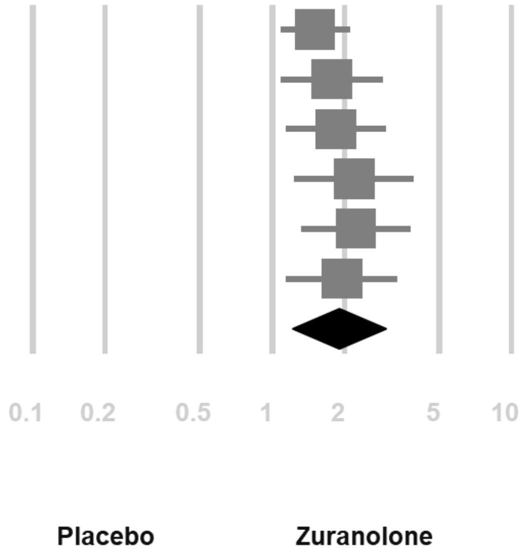

(D) Anxiety symptoms

Std diff in means (95%CI) with study removed

Study name	Point	Lower limit	Upper limit
Gunduz-Bruce (2019)	-0.20	-0.30	-0.10
Deligiannidis (2021)	-0.22	-0.35	-0.09
Kato (2023)	-0.27	-0.42	-0.11
Clayton (2023a)	-0.29	-0.42	-0.16
Clayton (2023b)	-0.28	-0.45	-0.11
Deligiannidis (2023)	-0.24	-0.39	-0.09
Pooled	-0.25	-0.37	-0.12

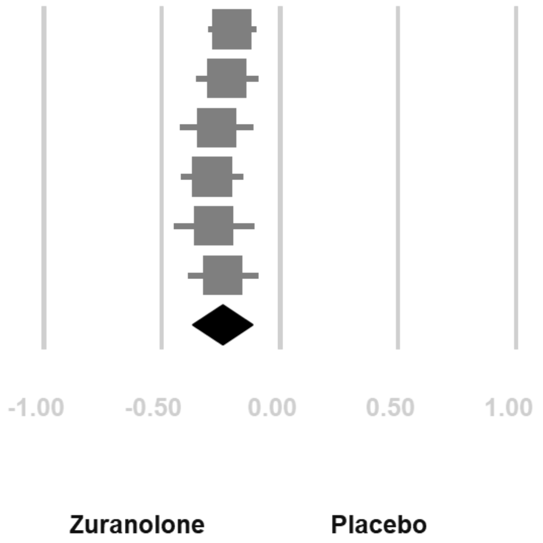

(E) Dropout rate

Study name	Point	Lower limit	Upper limit
NCT03771664	1.11	0.84	1.47
Gunduz-Bruce (2019)	1.15	0.86	1.52
Deligiannidis (2021)	1.17	0.88	1.55
Kato (2023)	1.12	0.83	1.50
Clayton (2023a)	1.11	0.75	1.64
Clayton (2023b)	1.12	0.82	1.52
Deligiannidis (2023)	1.11	0.84	1.49
Pooled	1.13	0.85	1.49

Odds ratio (95% CI) with study removed

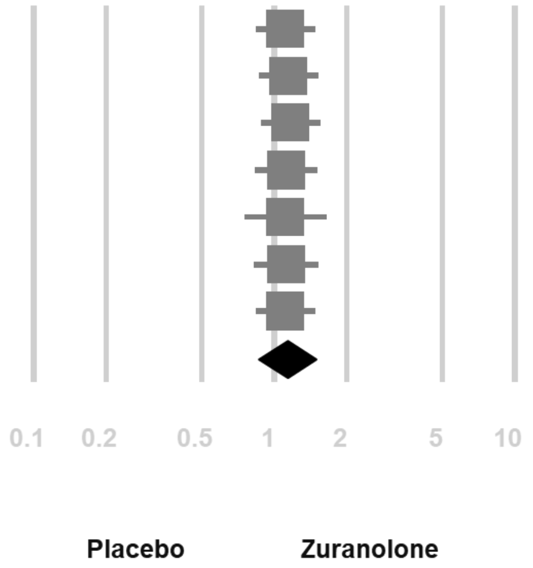

(F) Any side effect rate

Study name	Point	Lower limit	Upper limit
NCT03771664	1.48	1.22	1.79
Gunduz-Bruce (2019)	1.39	1.09	1.77
Deligiannidis (2021)	1.39	1.08	1.78
Kato (2023)	1.46	1.16	1.84
Clayton (2023a)	1.43	1.09	1.87
Clayton (2023b)	1.27	1.01	1.58
Deligiannidis (2023)	1.35	1.06	1.72
Pooled	1.40	1.14	1.74

Odds ratio (95% CI) with study removed

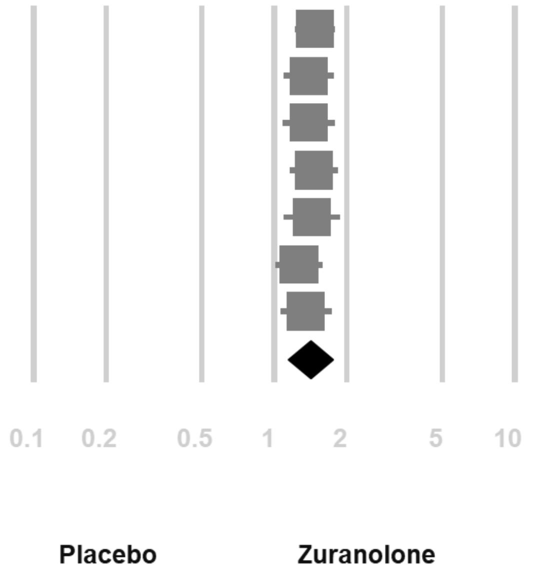

eFigure 3. Sensitivity analysis at fixed percentiles of 10%, 75%, and 95%

(A) Depressive symptoms

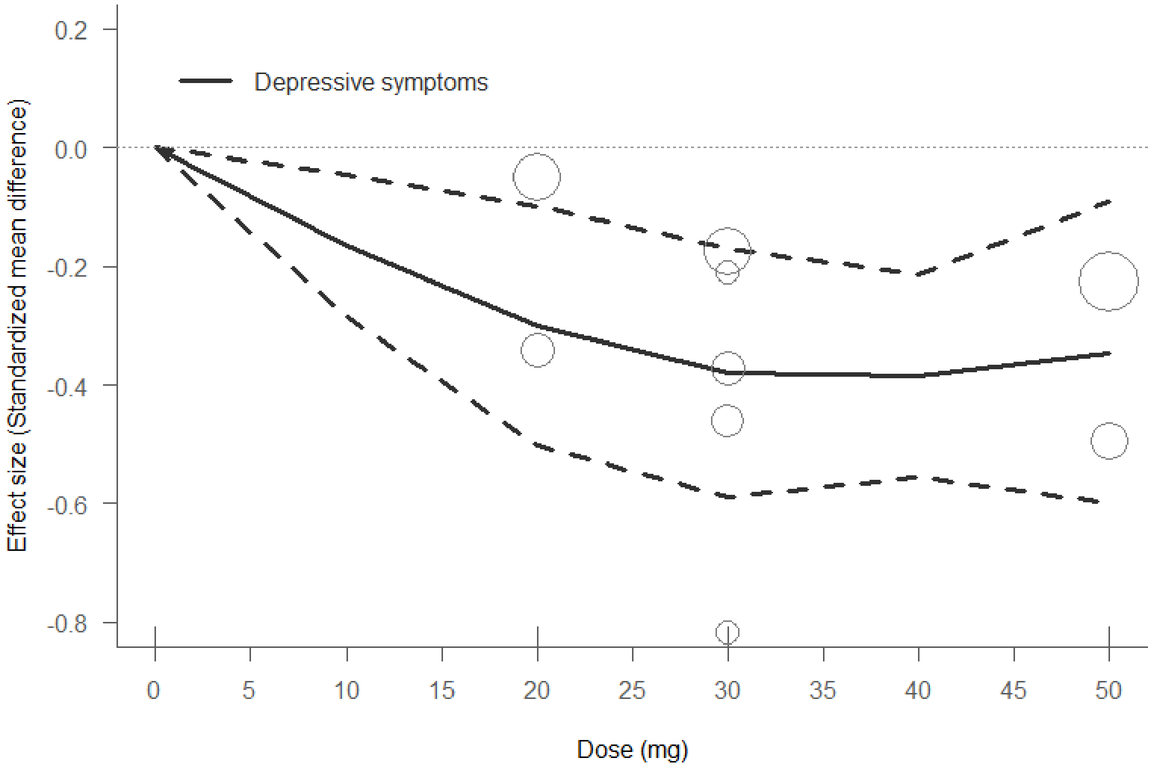

(B) Depression response rate

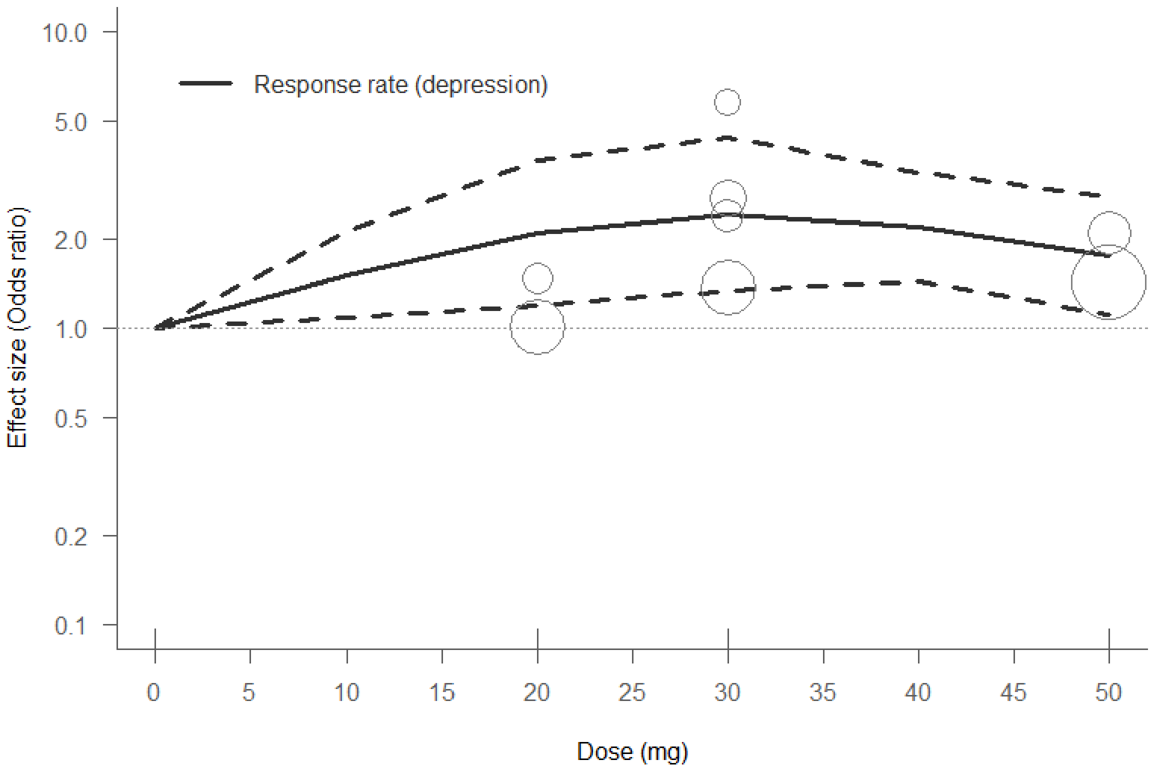

(C) Depression remission rate

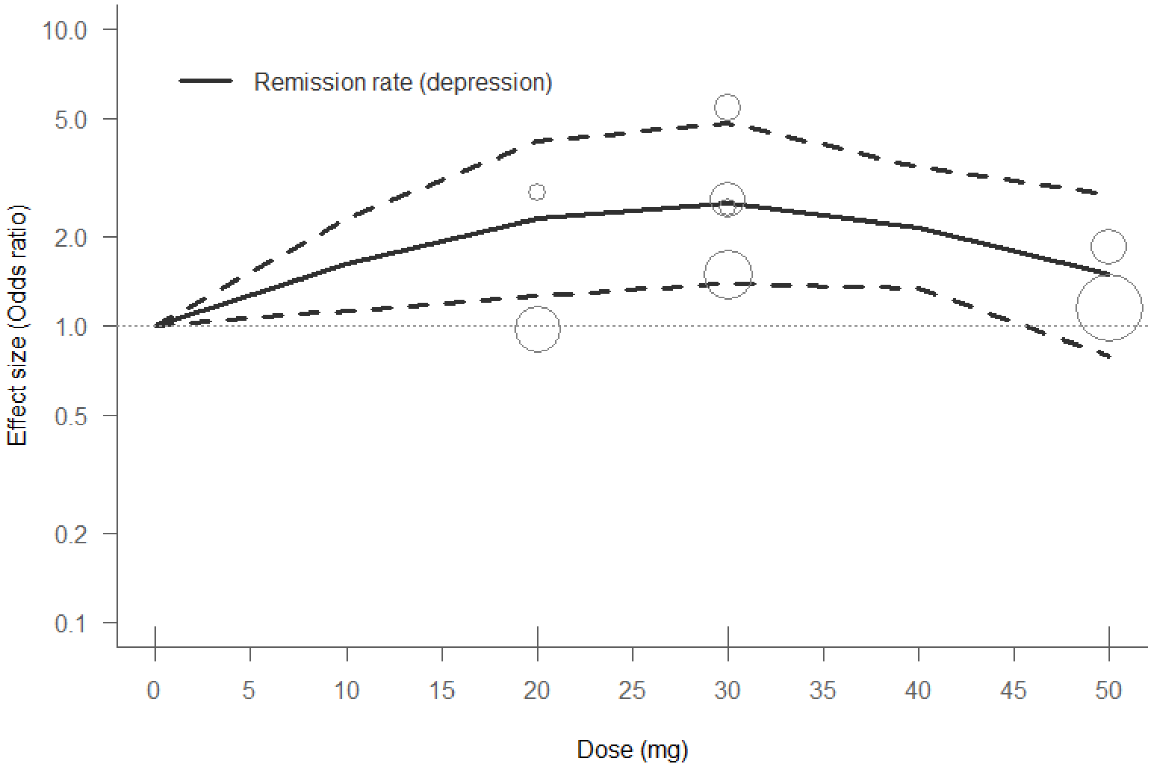

(D) Anxiety symptoms

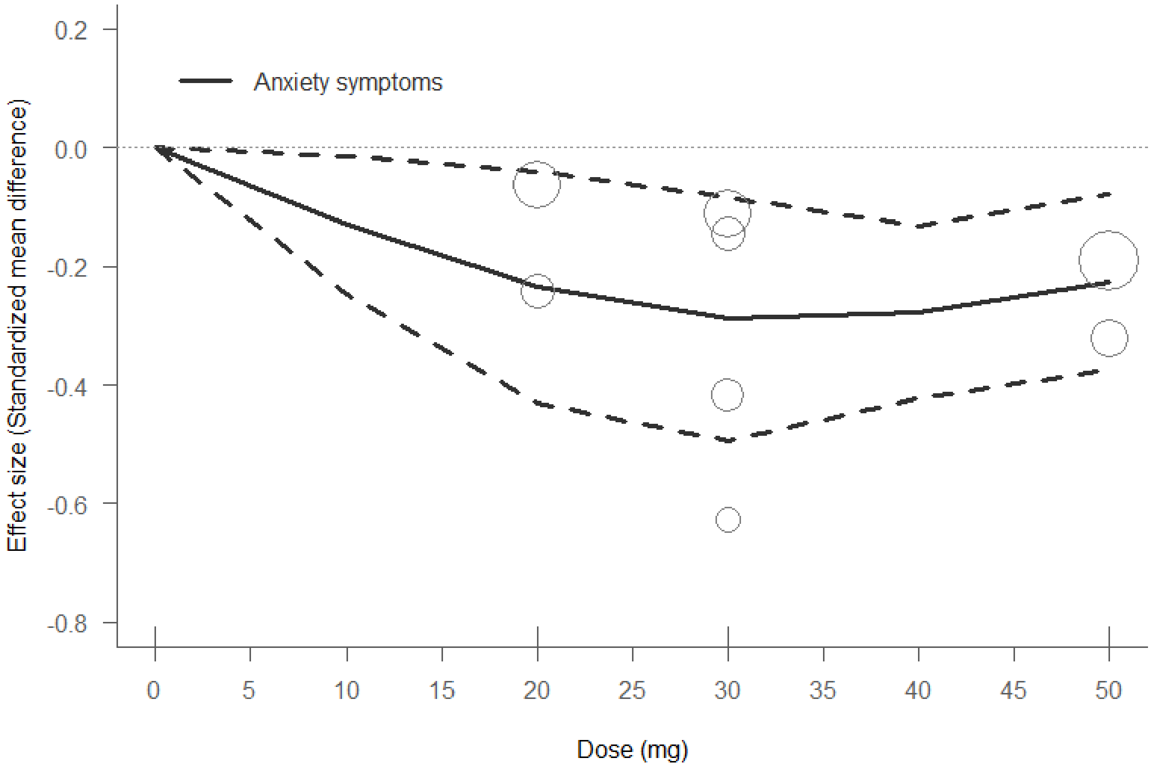

(E) Dropout rate

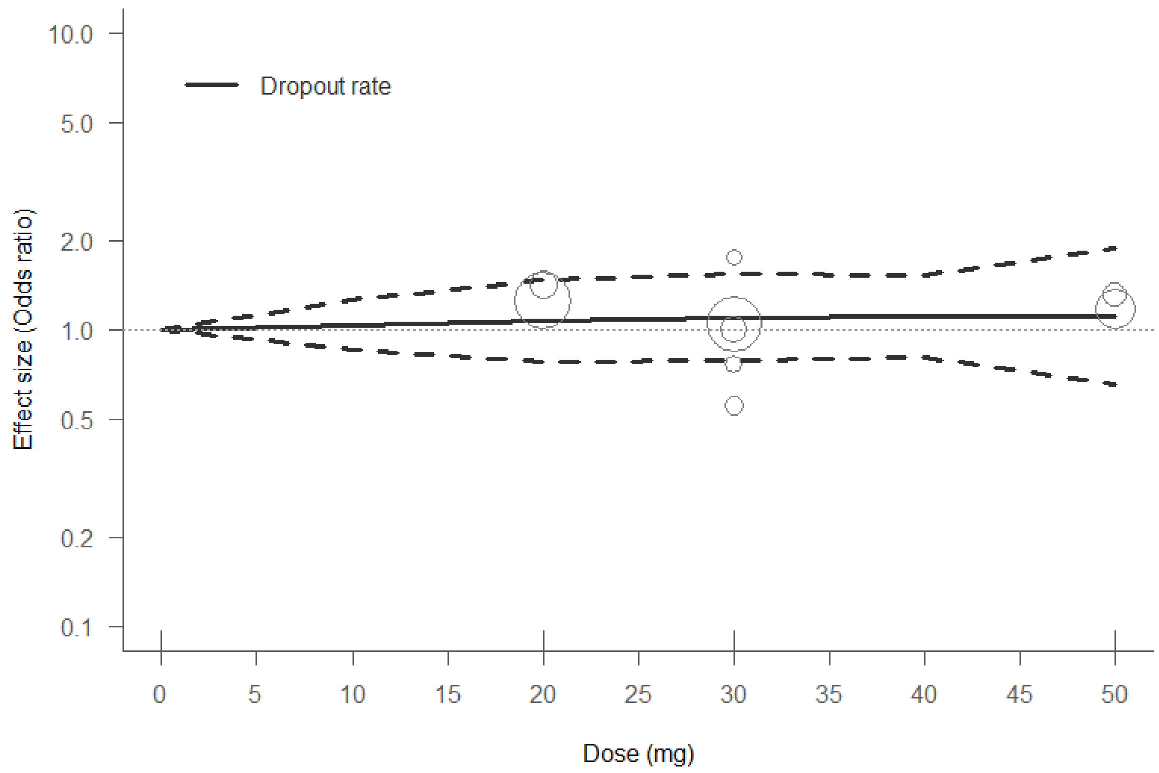

(F) Any side effect rate

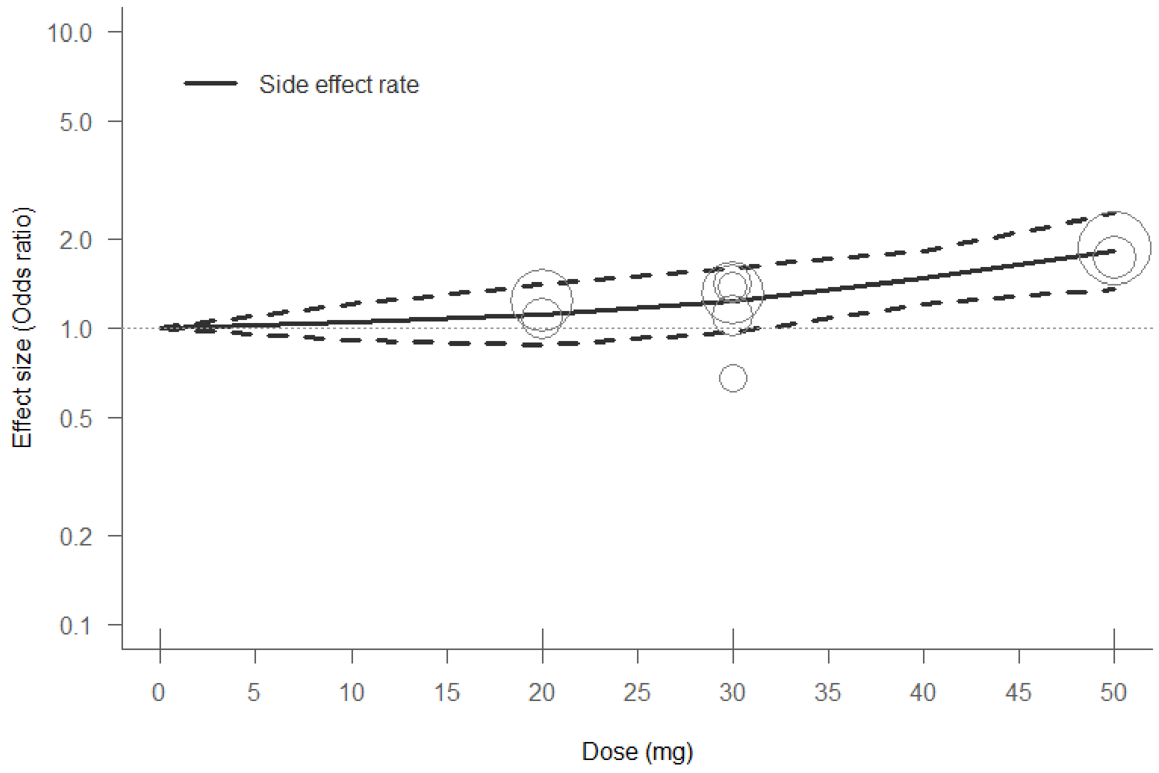

eFigure 4. Summary of quality assessment of included studies using Cochrane risk of bias 2 tool

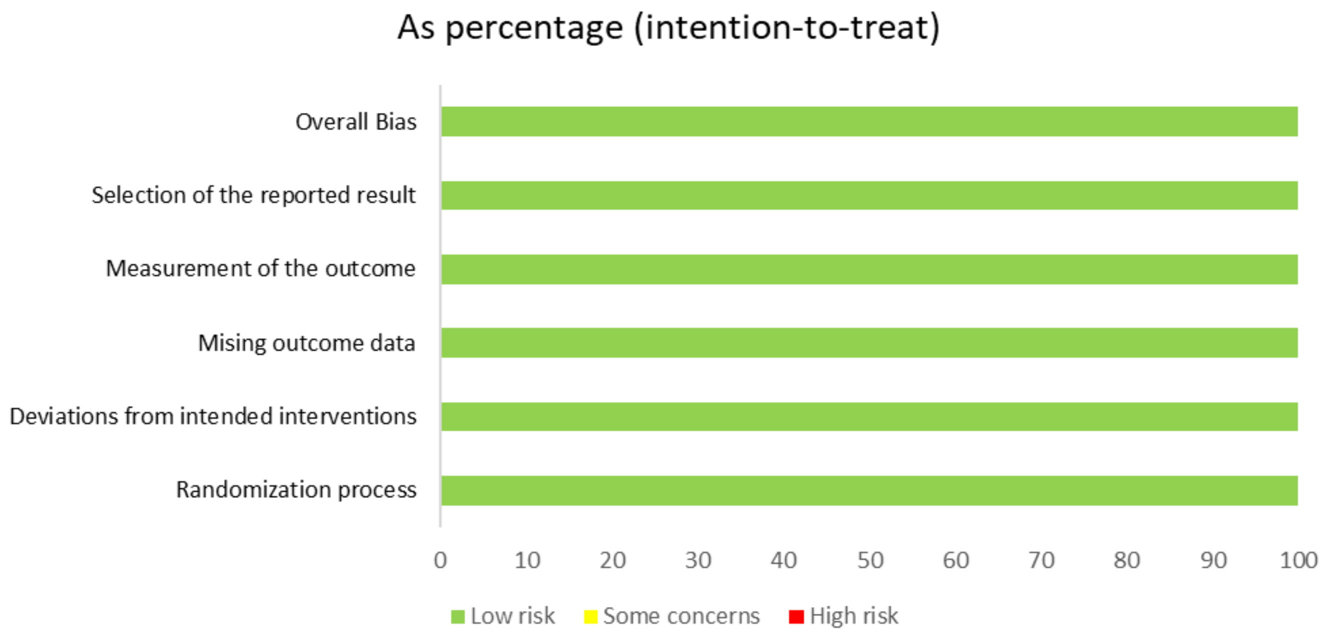

Study ID	D1	D2	D3	D4	D5	Overall
NCT03771664	+	+	+	+	+	+
Gunduz-Bruce 2019	+	+	+	+	+	+
Deligiannidis 2021	+	+	+	+	+	+
Clayton 2023a	+	+	+	+	+	+
Clayton 2023b	+	+	+	+	+	+
Kato 2023	+	+	+	+	+	+
Deligiannidis 2023	+	+	+	+	+	+

+	Low risk
!	Some concerns
-	High risk
D1	Randomisation process
D2	Deviations from the intended interventions
D3	Missing outcome data
D4	Measurement of the outcome
D5	Selection of the reported result

eFigure 5. Funnel plots with Egger's test of meta-analysis

(A) Depressive symptoms

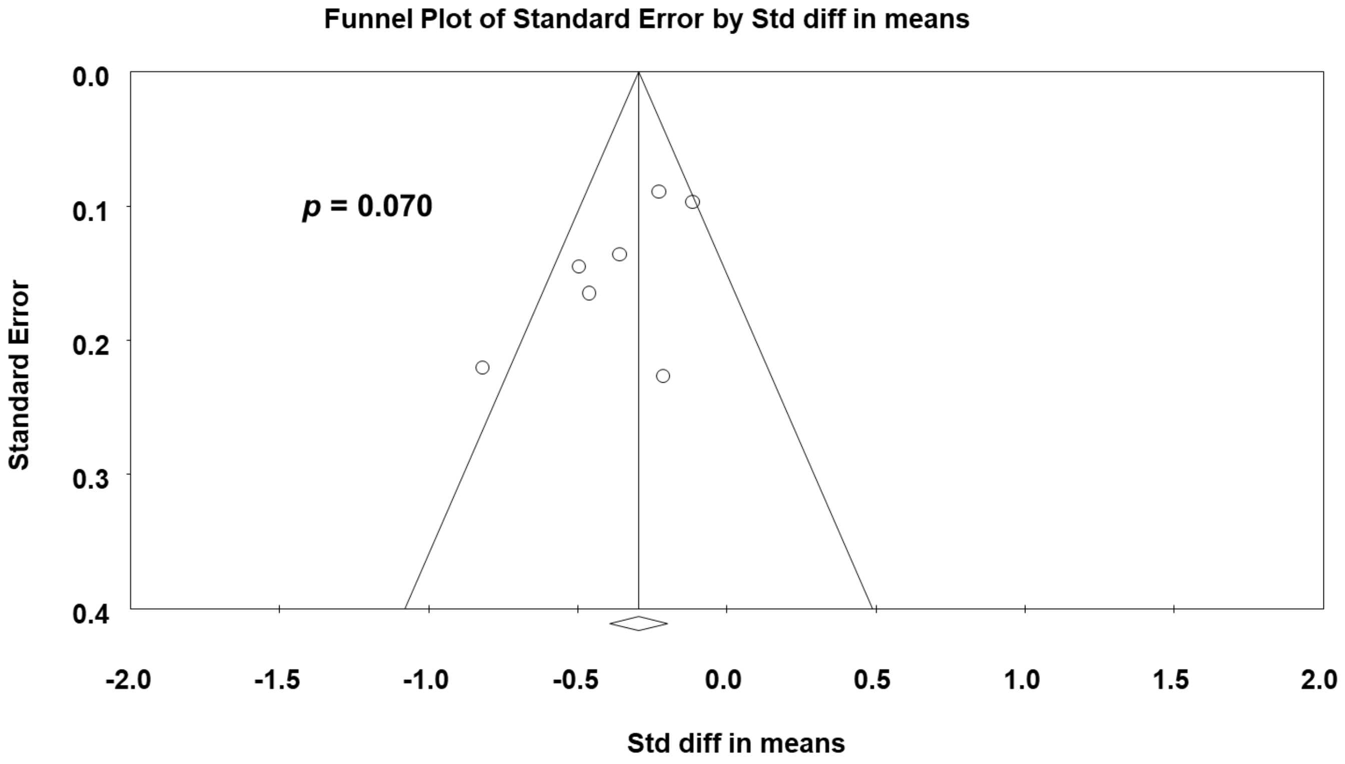

(B) Depression response rate

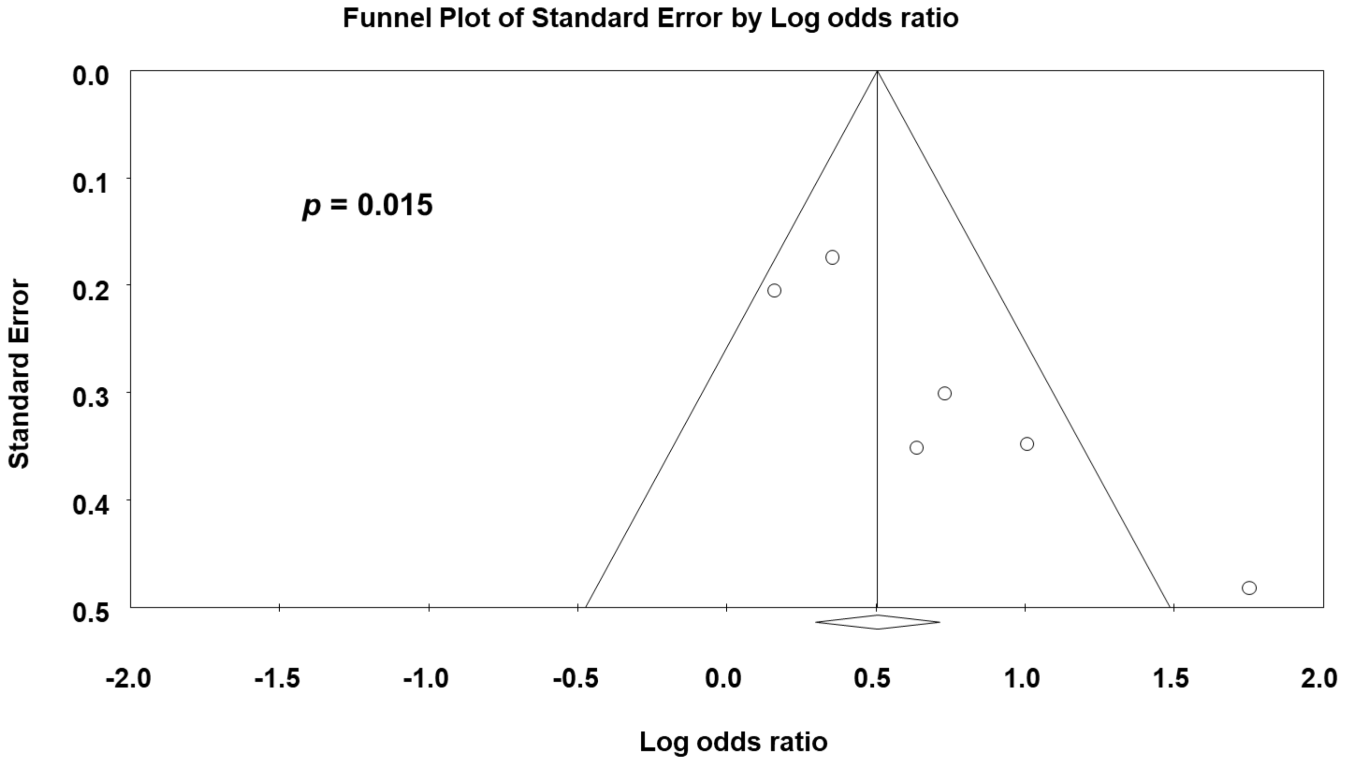

(C) Depression remission rate

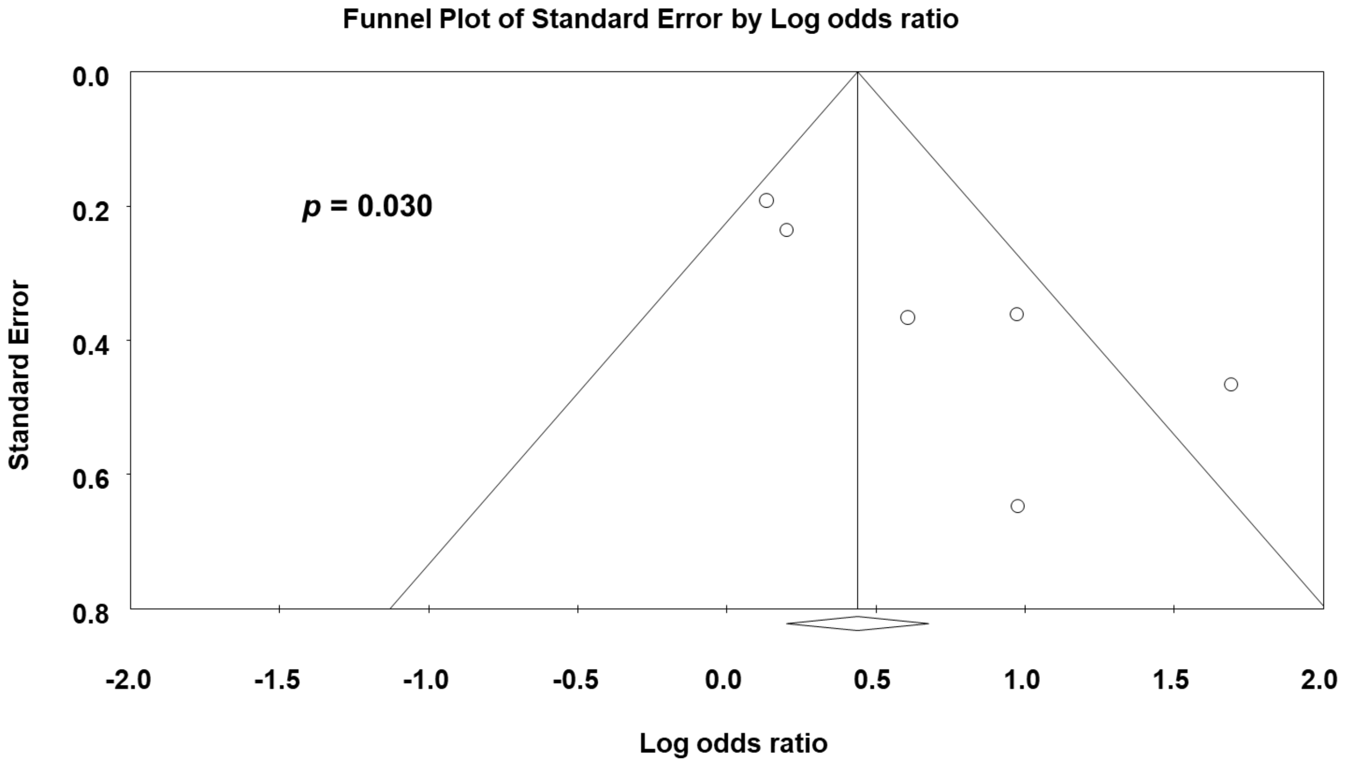

(D) Anxiety symptoms

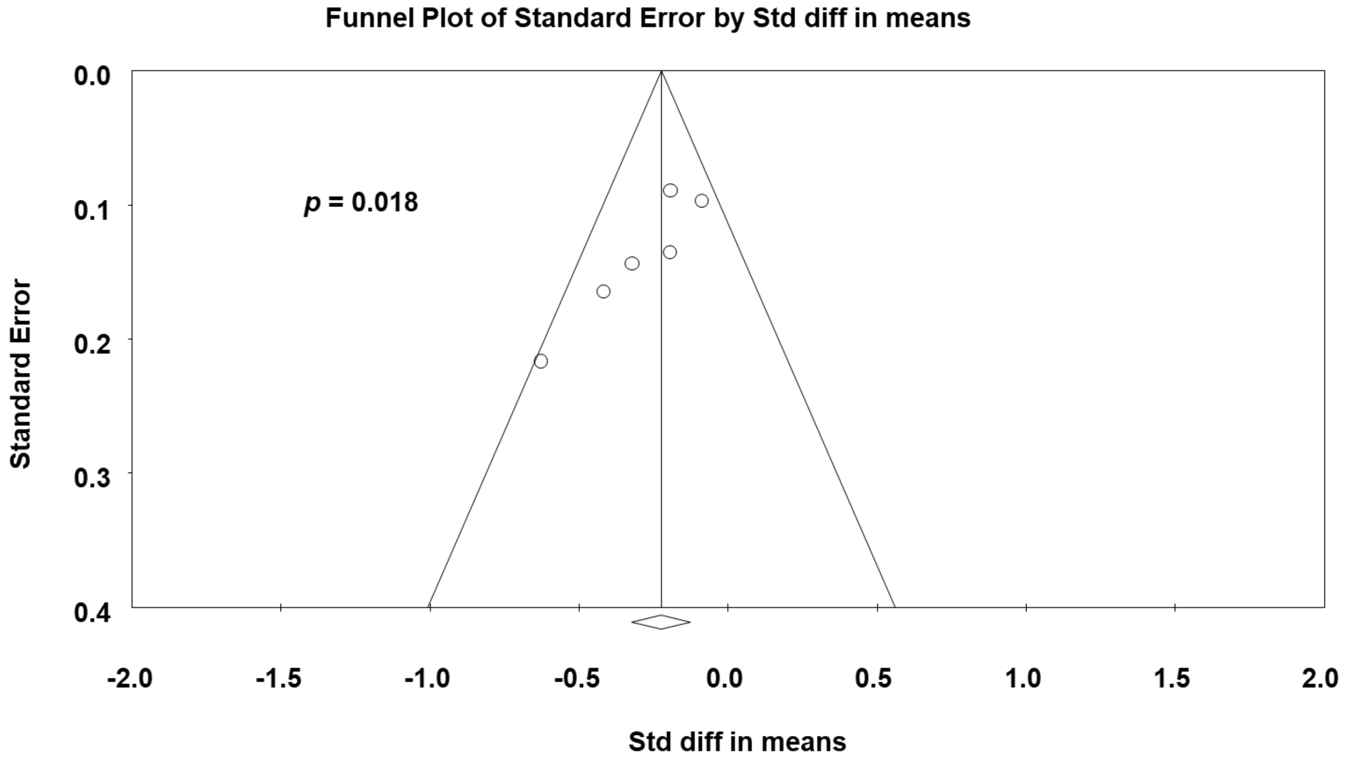

(E) Dropout rate

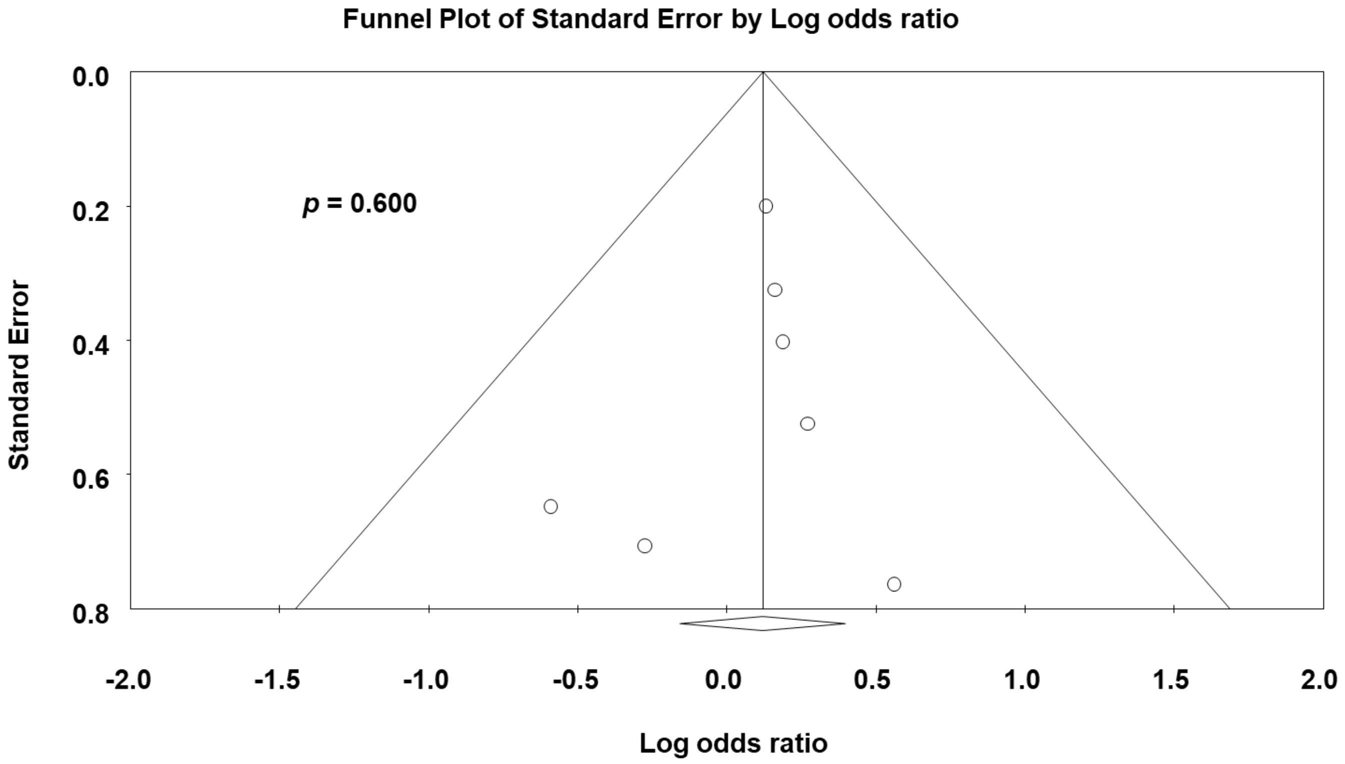

(F) Any side effect rate

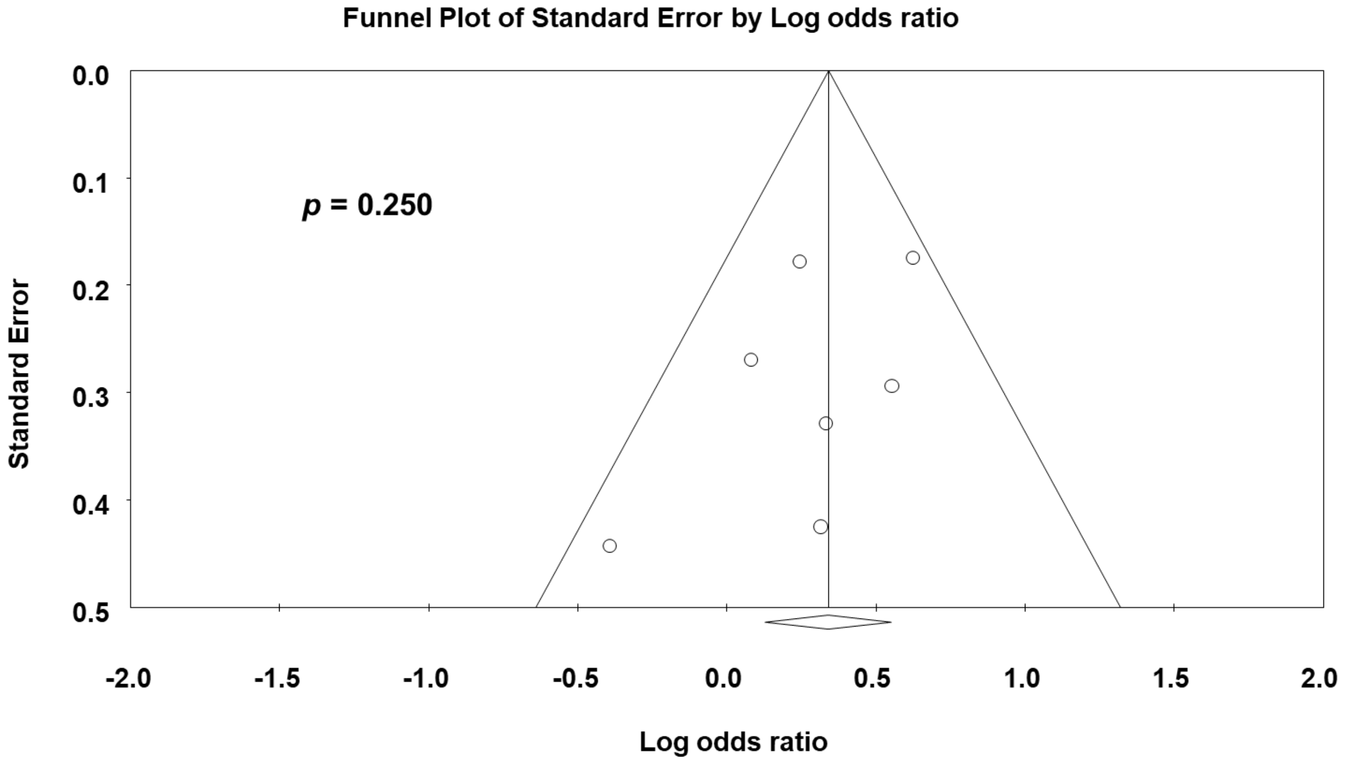

eFigure 6. Funnel plots after trim-and-fill method

(A) Depression response rate

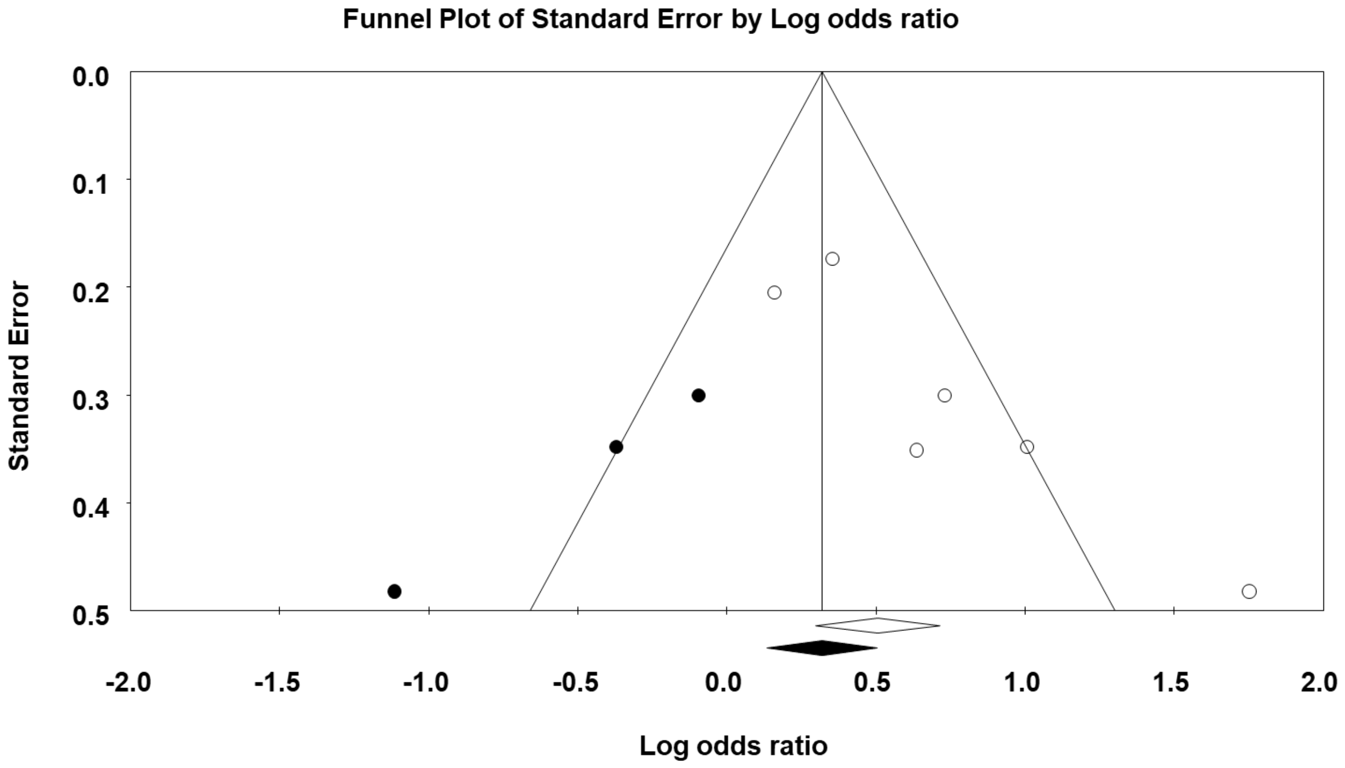

(B) Depression remission rate

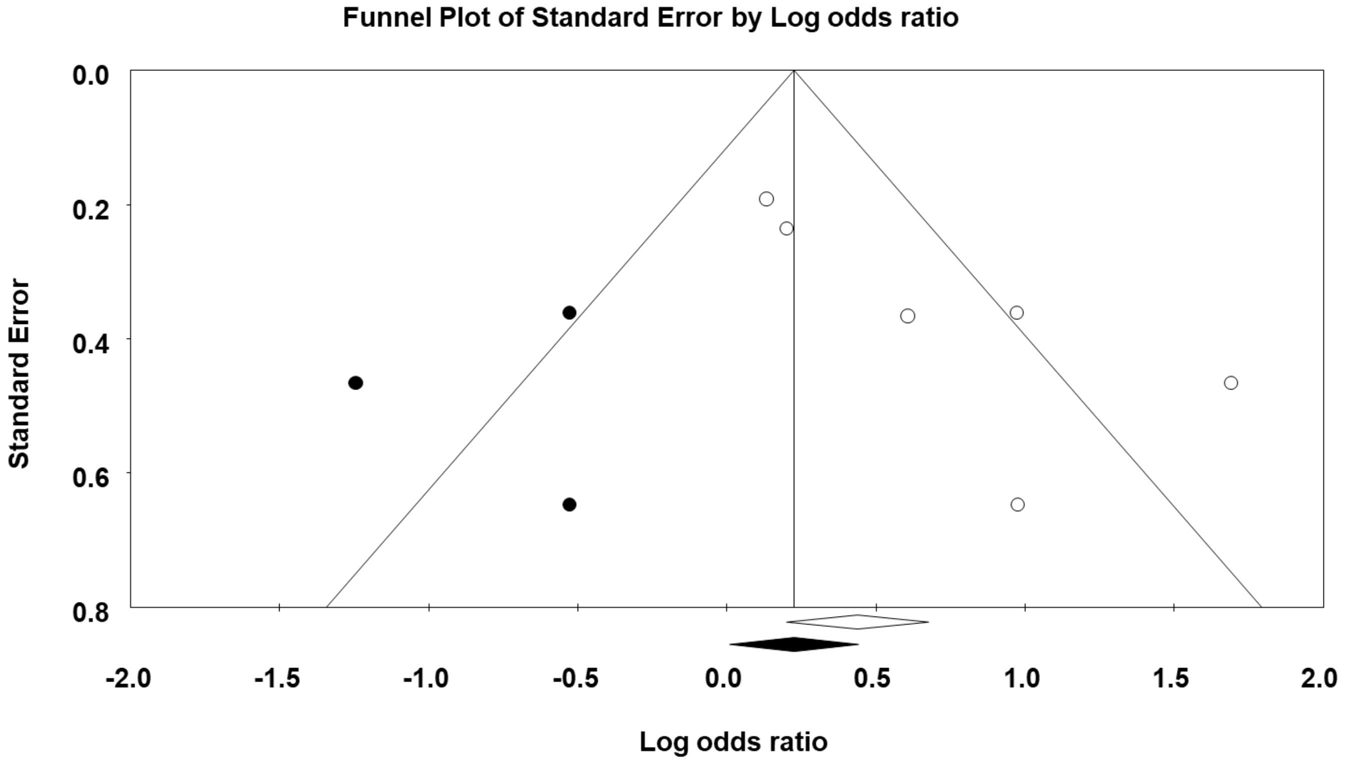

(C) Anxiety symptoms

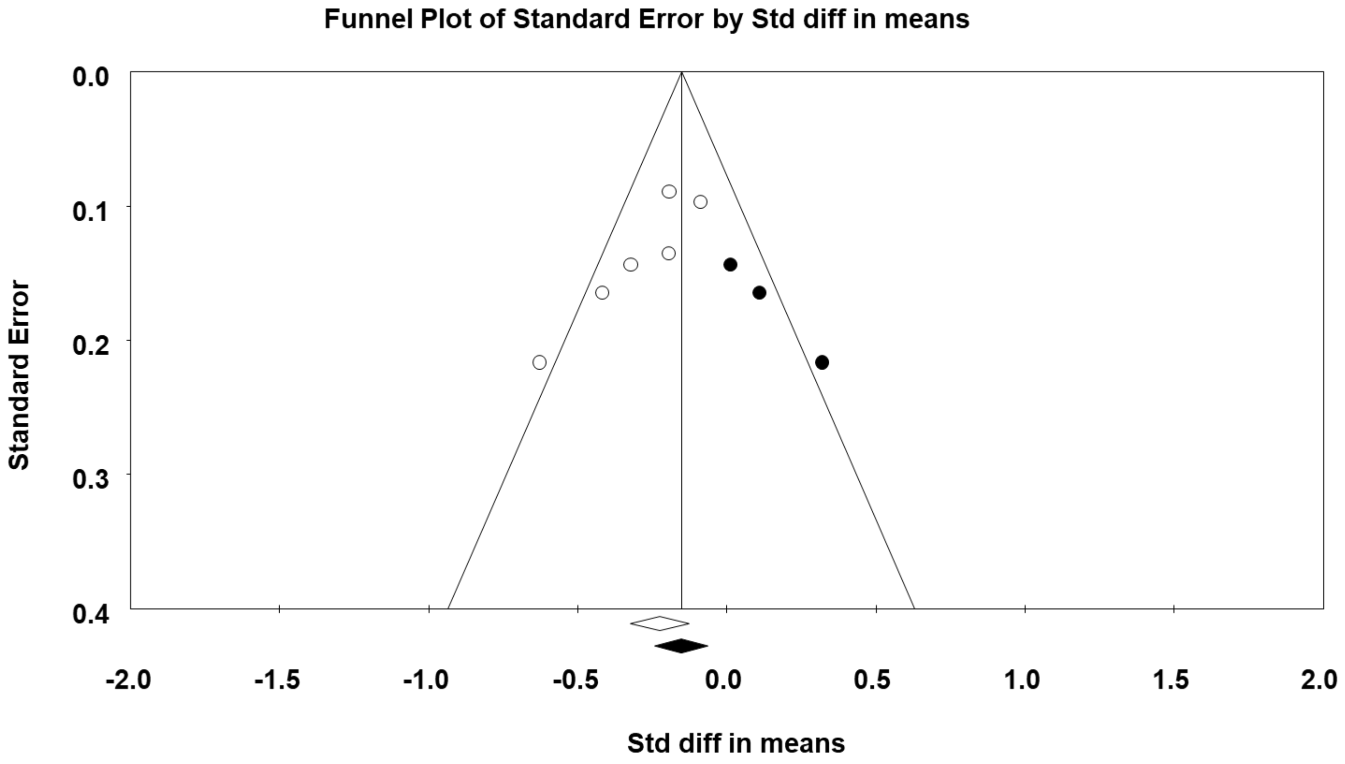

eFigure 7. Variation partition coefficients of dose-response meta-analysis

(A) Depressive symptoms

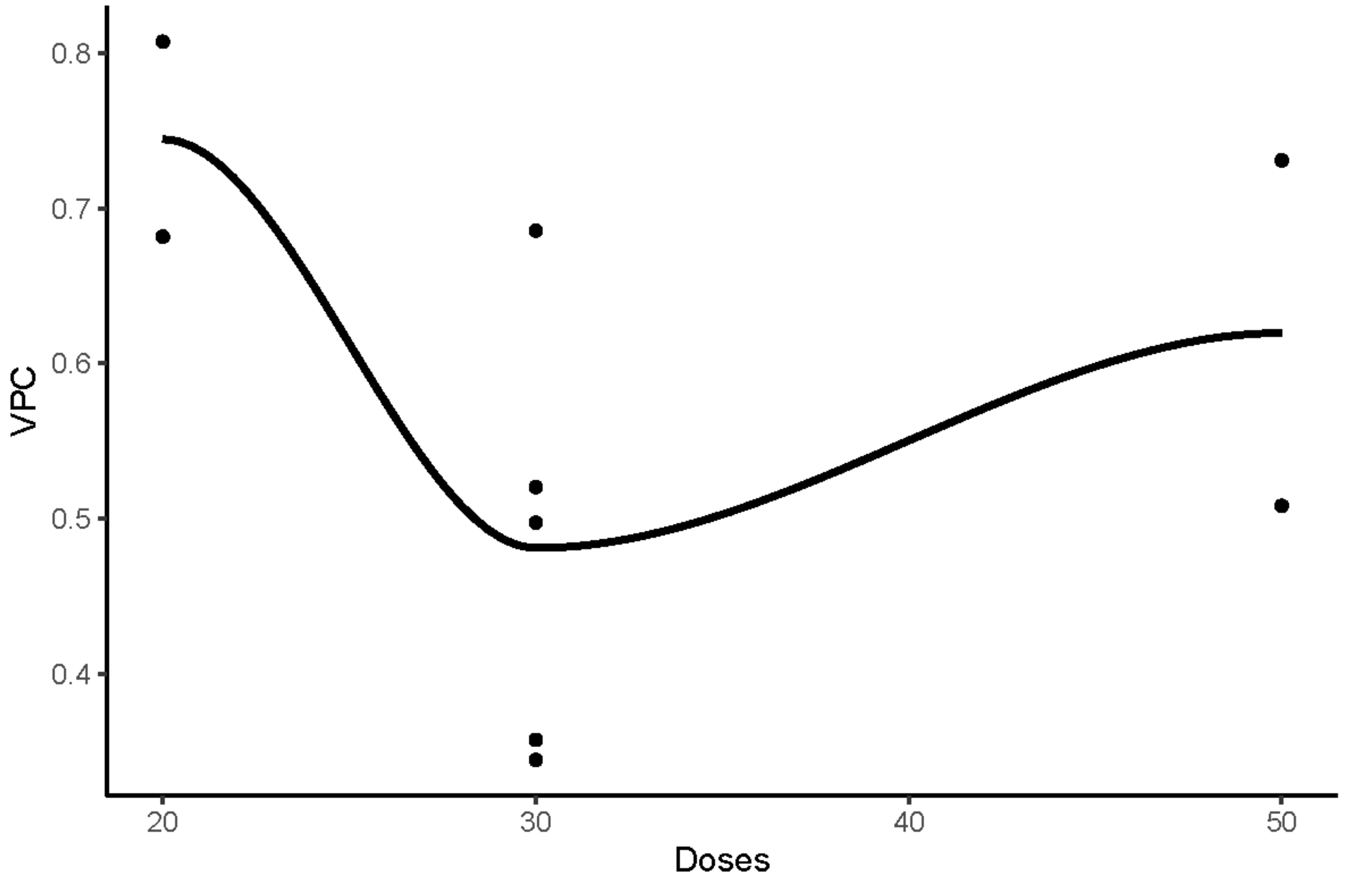

(B) Depression response rate

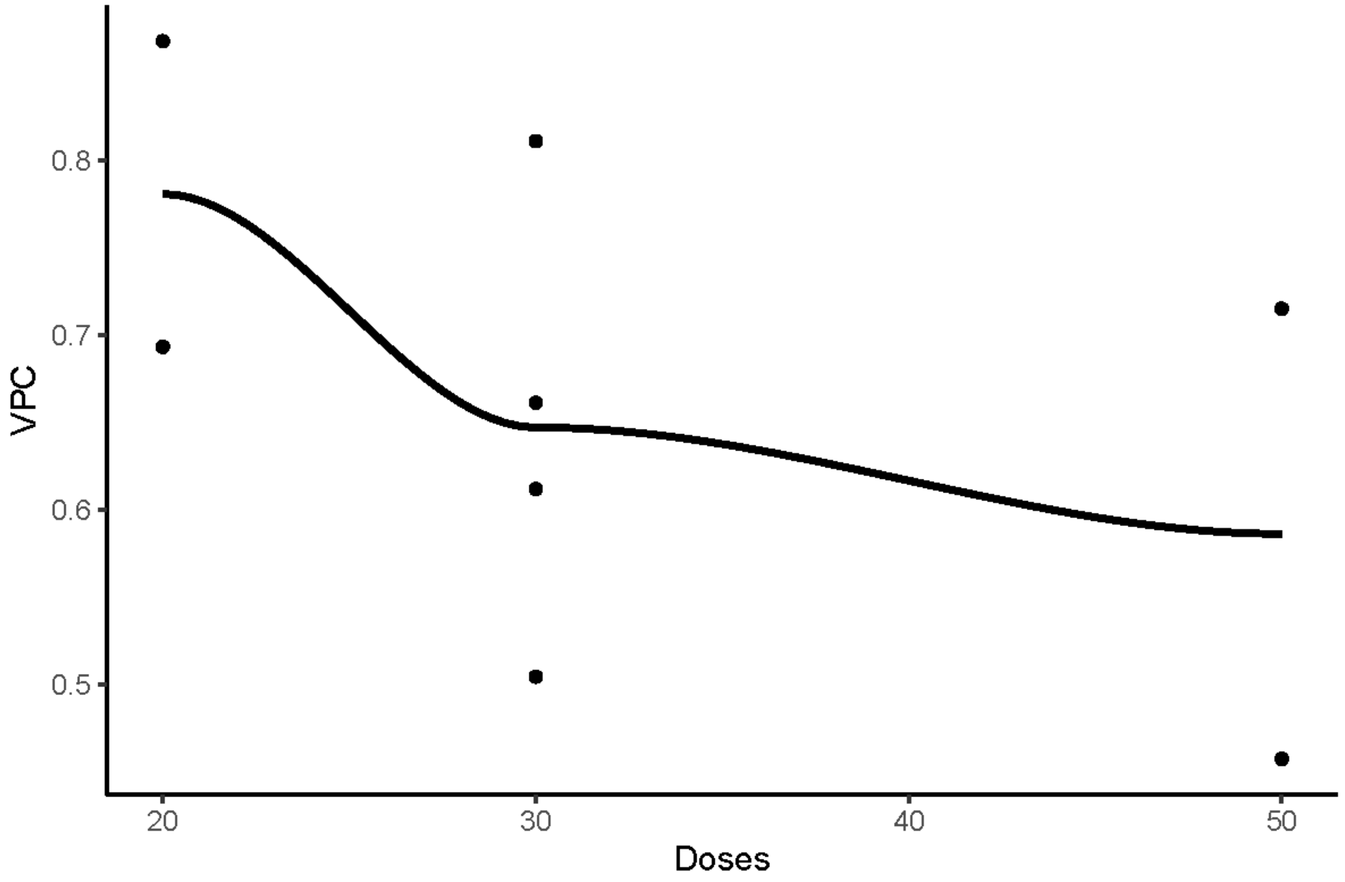

(C) Depression remission rate

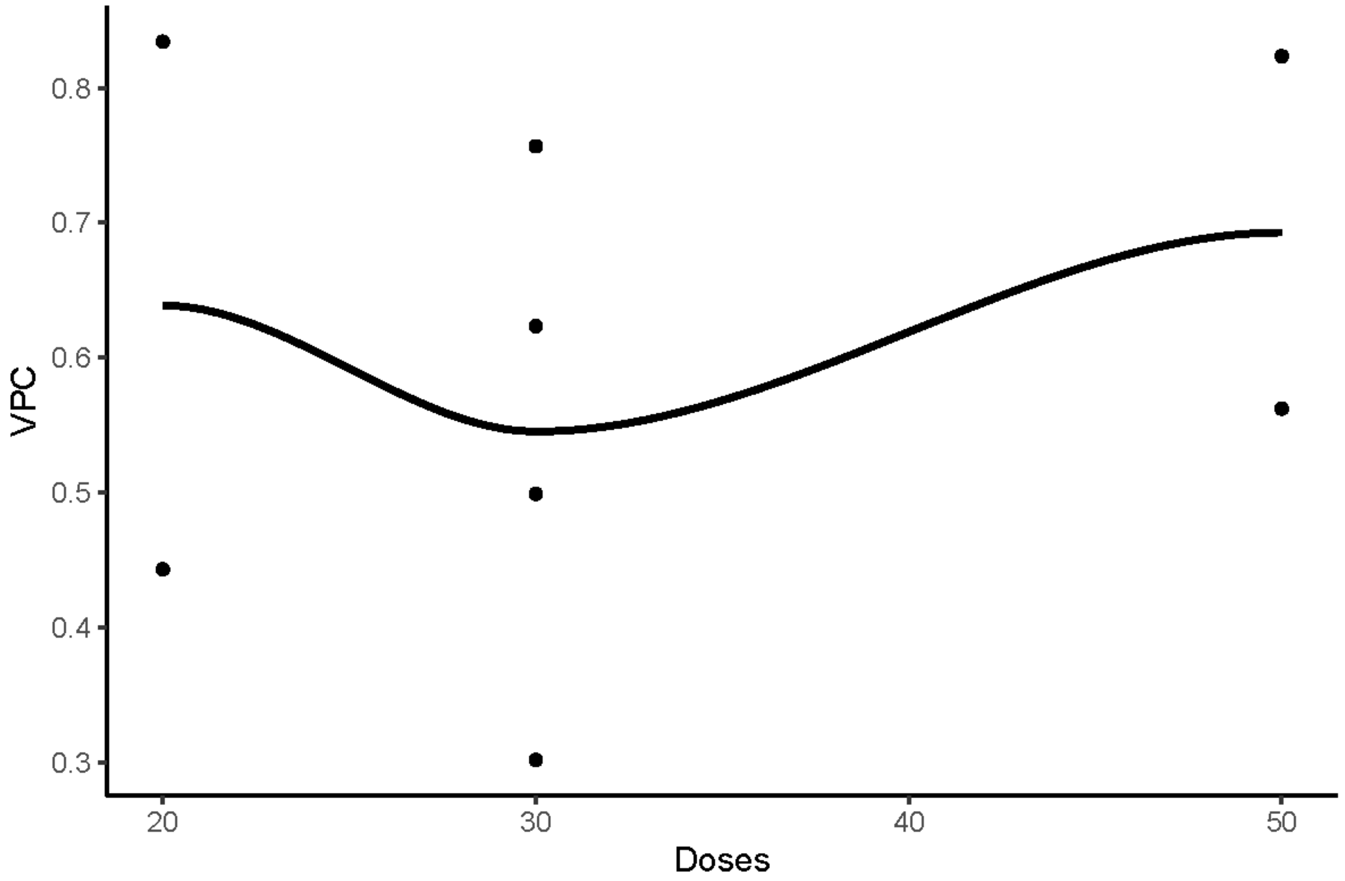

(D) Anxiety symptoms

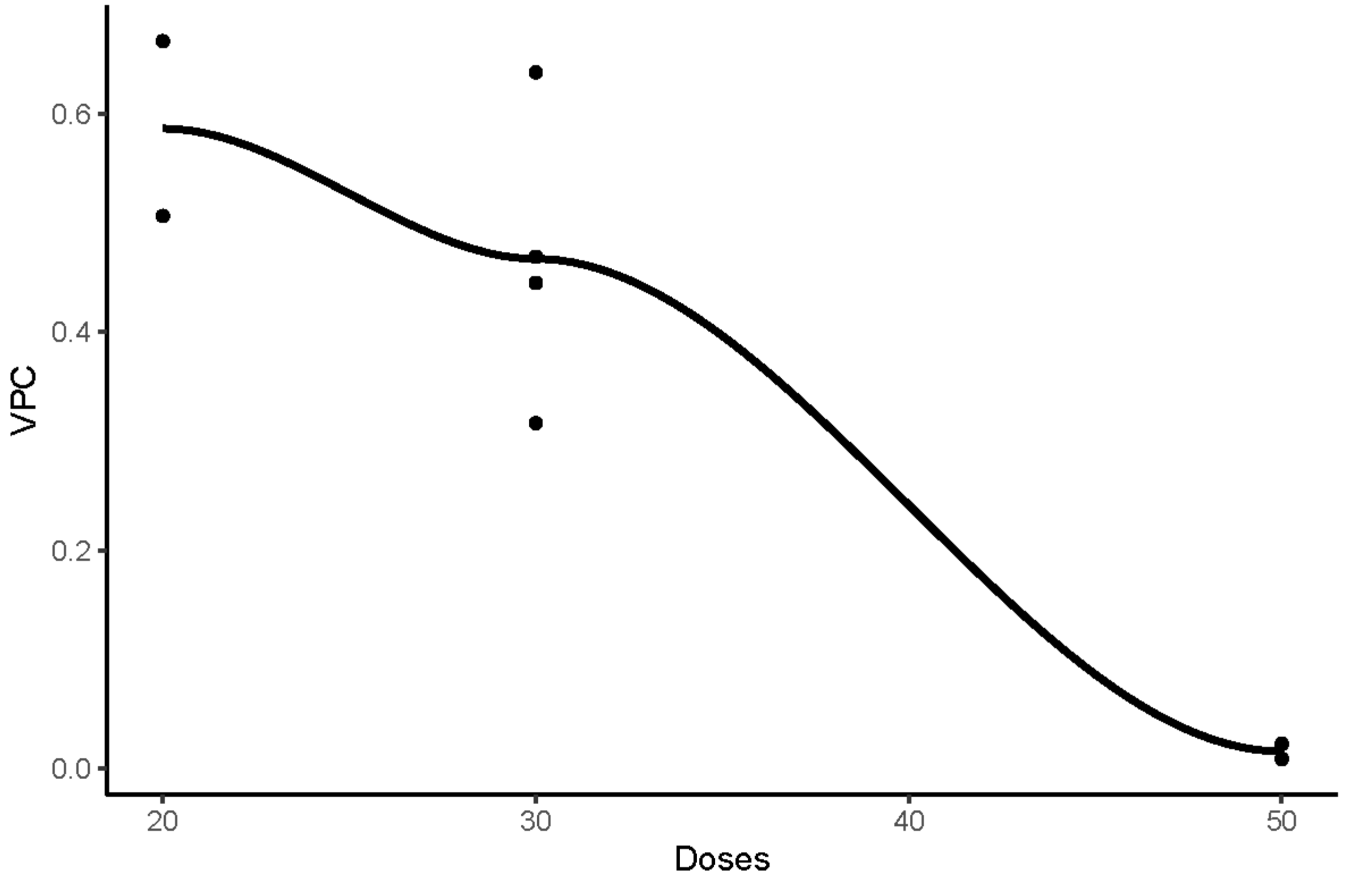

(E) Dropout rate

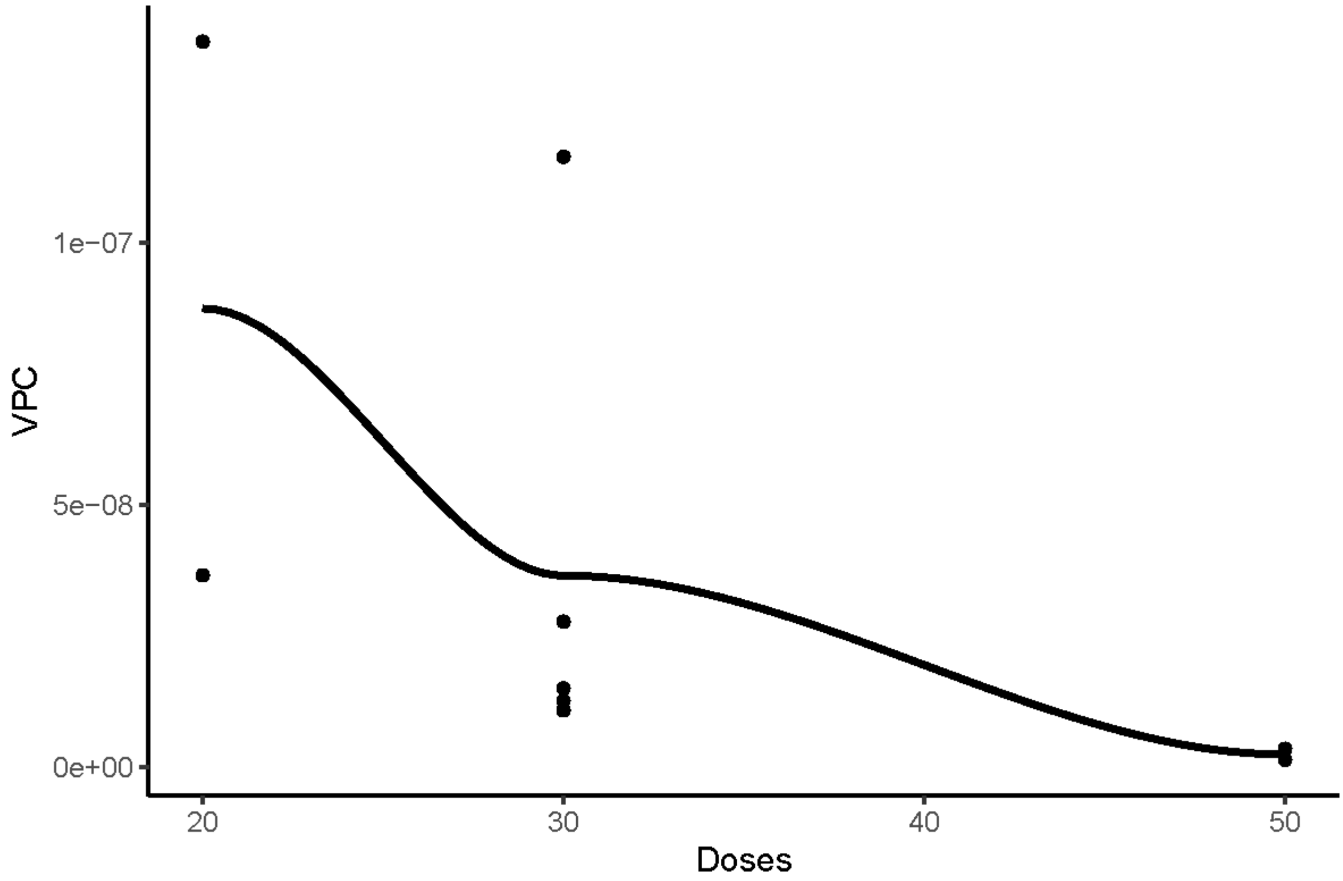

(F) Any side effect rate

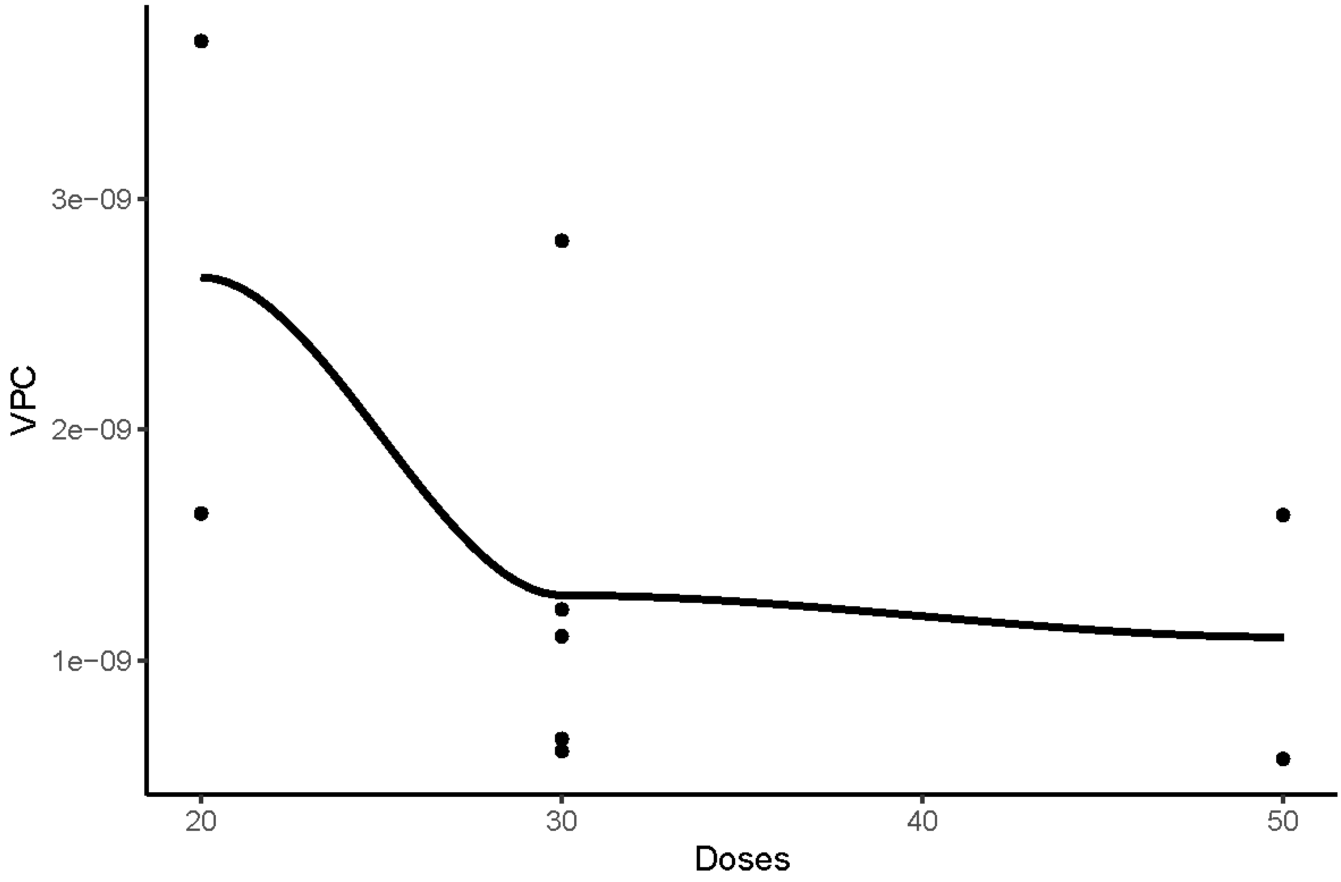

References

1. Gunduz-Bruce H, Silber C, Kaul I, et al. Trial of SAGE-217 in Patients with Major Depressive Disorder. New England Journal of Medicine 2019;381:903-11.
2. Deligiannidis KM, Meltzer-Brody S, Gunduz-Bruce H, et al. Effect of Zuranolone vs Placebo in Postpartum Depression A Randomized Clinical Trial. Jama Psychiatry 2021;78:951-9.
3. Kato M, Nakagome K, Baba T, et al. Efficacy and safety of zuranolone in Japanese adults with major depressive disorder: A double-blind, randomized, placebo-controlled, phase 2 clinical trial. Psychiatry and clinical neurosciences 2023.
4. Clayton AH, Lasser R, Nandy I, Sankoh AJ, Jonas J, Kanes SJ. Zuranolone in Major Depressive Disorder: Results From MOUNTAIN-A Phase 3, Multicenter, Double-Blind, Randomized, Placebo-Controlled Trial. J Clin Psychiatry 2023a;84.
5. Clayton AH, Lasser R, Parikh SV, et al. Zuranolone for the Treatment of Adults With Major Depressive Disorder: A Randomized, Placebo-Controlled Phase 3 Trial. Am J Psychiatry 2023b:appiajp20220459.
6. Deligiannidis KM, Meltzer-Brody S, Maximos B, et al. Zuranolone for the Treatment of Postpartum Depression. *Am J Psychiatry* 2023: appiajp20220785.
7. National Library of Medicine (U.S.). 2019 F-, Jan. A Study to Evaluate the Safety, Tolerability, and Efficacy of SAGE-217 Compared to Placebo in Adult Subjects With Comorbid Major Depressive Disorder and Insomnia. Identifier: NCT03771664. https://clinicaltrialsgov/show/NCT03771664.
